# MMP9 modulation improves specific neurobehavioral deficits in a mouse model of Alzheimer’s disease

**DOI:** 10.1186/s12868-021-00643-2

**Published:** 2021-05-25

**Authors:** Charis Ringland, Jonas Elias Schweig, Maxwell Eisenbaum, Daniel Paris, Ghania Ait-Ghezala, Michael Mullan, Fiona Crawford, Laila Abdullah, Corbin Bachmeier

**Affiliations:** 1 The Roskamp Institute, 2040 Whitfield Avenue, Sarasota, FL 34243 USA; 2 The Open University, Milton Keynes, UK; 3 James A. Haley Veterans’ Hospital, Tampa, FL USA; 4 Bay Pines VA Healthcare System, Bay Pines, FL USA

**Keywords:** Alzheimer’s disease, Matrix metallopeptidase 9, Apolipoprotein E, Social recognition memory, Beta-amyloid

## Abstract

**Background:**

Matrix metallopeptidase 9 (MMP9) has been implicated in a variety of neurological disorders, including Alzheimer’s disease (AD), where MMP9 levels are elevated in the brain and cerebrovasculature. Previously our group demonstrated apolipoprotein E4 (apoE4) was less efficient in regulating MMP9 activity in the brain than other apoE isoforms, and that MMP9 inhibition facilitated beta-amyloid (Aβ) elimination across the blood–brain barrier (BBB)

**Methods:**

In the current studies, we evaluated the impact of MMP9 modulation on Aβ disposition and neurobehavior in AD using two approaches, (1) pharmacological inhibition of MMP9 with SB-3CT in apoE4 x AD (E4FAD) mice, and (2) gene deletion of MMP9 in AD mice (MMP9KO/5xFAD)

**Results:**

Treatment with the MMP9 inhibitor SB-3CT in E4FAD mice led to reduced anxiety compared to placebo using the elevated plus maze. Deletion of the MMP9 gene in 5xFAD mice also reduced anxiety using the open field test, in addition to improving sociability and social recognition memory, particularly in male mice, as assessed through the three-chamber task, indicating certain behavioral alterations in AD may be mediated by MMP9. However, neither pharmacological inhibition of MMP9 or gene deletion of MMP9 affected spatial learning or memory in the AD animals, as determined through the radial arm water maze. Moreover, the effect of MMP9 modulation on AD neurobehavior was not due to changes in Aβ disposition, as both brain and plasma Aβ levels were unchanged in the SB-3CT-treated E4FAD animals and MMP9KO/AD mice compared to their respective controls.

**Conclusions:**

In total, while MMP9 inhibition did improve specific neurobehavioral deficits associated with AD, such as anxiety and social recognition memory, modulation of MMP9 did not alter spatial learning and memory or Aβ tissue levels in AD animals. While targeting MMP9 may represent a therapeutic strategy to mitigate aspects of neurobehavioral decline in AD, further work is necessary to understand the nature of the relationship between MMP9 activity and neurological dysfunction.

**Supplementary Information:**

The online version contains supplementary material available at 10.1186/s12868-021-00643-2.

## Background

Matrix metalloprotease 9 (MMP9) is a type IV collagenase and a member of the endopeptidase family of proteins, which contribute to tissue remodeling by degrading extracellular matrix components. MMP9 is expressed in a variety of cell types within the brain and is released from the cell as a pro-MMP9 precursor, before being activated extracellularly [123]. MMP9 has been implicated in a variety of neurological and inflammatory disease states and elevated MMP9 levels have been reported in cerebrovascular disorders such as Alzheimer’s disease (AD) [1–3], cerebral amyloid angiopathy [4], ischemia [5, 6], and intracerebral hemorrhage [7]. With respect to AD, studies have shown that MMP9 brain levels were elevated in patients with moderate and late AD [8] and MMP9 levels in cerebrospinal fluid (CSF) were associated with faster decline in an MCI to AD conversion group [9]. In other words, in these studies, higher levels of MMP9 in the brain and CSF were associated with later-stage AD [8] and correlated with declines in hippocampal volume and cognitive function [9]. Moreover, MMP9 has been found to play a causal role in amyloid β (Aβ)-induced cognitive impairment and neurotoxicity [10], as intracerebroventricular injection of Aβ induced cognitive deficits and MMP9 expression in the mouse hippocampus, while Aβ exposure caused neurotoxicity in cultured neurons. Importantly, the Aβ-induced cognitive impairment in vivo as well as neurotoxicity in vitro was significantly alleviated in MMP-9 homozygous knockout (KO) mice and treatment with MMP inhibitors [10].

Apolipoprotein E (APOE) is a major genetic risk factor for AD and has been shown by our group and others to influence Aβ clearance from the brain in an isoform-specific manner, with apoE4 being associated with reduced blood–brain barrier (BBB) transit from the brain [11–15]. Our team also found an isoform-specific effect of apoE on the ectodomain shedding of low-density lipoprotein receptor (LDLR) and LDLR-related protein 1 (LRP1) in brain endothelia with a rank order of apoE4 > apoE3 > apoE2 [16]. Importantly, these findings showed an inverse relationship between lipoprotein receptor shedding in the brain and Aβ elimination across the BBB, one that is APOE-genotype specific [16]. Our prior studies suggest the effect of apoE on these processes may be mediated through MMP9 [17, 18]. Based on in vitro and ex vivo studies, MMP9 is able to bind and proteolyse lipoprotein receptors prompting ectodomain shedding [19–22]. The proteolytic shedding of LRP1 and LDLR by MMP9 impairs their ability to transport Aβ out of the brain [16, 17]. Previously, we have shown that apoE influences MMP9 disposition in the brain in an isoform-dependent manner [18]. We demonstrated that apoE influences MMP9 levels in brain endothelia, the conversion of proMMP9 to active MMP9, and dose-dependently inhibits MMP9 activity. Importantly, apoE4 was the least effective in modulating these processes compared to other apoE isoforms, which may be due to a weaker binding affinity to MMP9 [18]. Furthermore, with respect to AD, MMP9 levels were significantly elevated in the cerebrovasculature of both human and animal AD brain specimens with an APOE4 genotype [18].

It has been demonstrated that MMP9 knockout (MMP9KO) mice were protected against cerebral ischemia [23] and traumatic brain injury [24], reportedly due to the prevention of MMP9 activity at the BBB. Pharmacologic inhibition of MMPs has been shown to attenuate tissue damage and edema in cerebral focal ischemia [25, 26] and ameliorate neutrophil infiltration, oxidative stress, edema and degenerating neurons in intracerebral hemorrhage [7]. In a transgenic mouse model of AD, minocycline treatment diminished inducible nitric oxide synthase and activation of microglia whilst ameliorating cognitive dysfunction which was attributed in part to the inhibition of MMP9 [27]. Lastly, intracranial injection of Aβ increased MMP9 expression and induced hippocampal damage alongside learning and memory deficits [10, 28–30], which was alleviated by MMP9 inhibitors and diminished in MMP9KO mice [10].

Despite its implications in AD and other neurological disorders, MMP9 has not been formally investigated as a target in AD models. The elevated MMP9 levels in AD brains and the reduced ability of apoE4 to modulate MMP9 disposition in our prior studies [18] prompted us to investigate the therapeutic value of modulating MMP9 activity in E4FAD transgenic animals, an AD mouse model expressing five familial AD (FAD) mutations (5xFAD mice) and homozygous for the human APOE4 gene. To assess the impact of modulating MMP9 activity on the AD phenotype, we used SB-3CT which has been recognized as a selective MMP-2 and MMP9 inhibitor [6, 31–33]. This compound readily crosses the BBB and was developed to circumvent the adverse side-effects associated with broad-spectrum MMP inhibitors [34, 35]. In addition to pharmacological inhibition of MMP9 in AD animals, we generated 5xFAD/MMP9 knockout mice (MMP9KO mice crossed with 5xFAD mice) as a complementary approach to evaluating the effect of MMP9 modulation on AD pathology and neurobehavior. Specifically, in the following studies we assessed the influence of pharmacological MMP9 inhibition and MMP9 gene knockout on amyloid pathology and lipoprotein receptor shedding in the brain alongside several measures of behaviour including anxiety, sociability, social recognition memory, and spatial learning and memory.

## Methods

### Materials

The MMP9 inhibitor, SB-3CT (2-[[(4-Phenoxyphenyl)sulfonyl]methyl]thiirane), was purchased from Tocris Bioscience (Minneapolis, MN, USA). Enzyme linked immunosorbent assay (ELISA) kits for mouse MMP9 were purchased from Sciencell Research Laboratories (Carlsbad, CA, USA). ELISA kits for LDLR and LRP1 receptors were purchased from Cedarlane Labs (Burlington, NC, USA). Halt enzyme inhibitor cocktails, the bicinchoninic acid (BCA) protein assay, Hanks Balanced Salt Solution (HBSS) and ELISA kits for human Aβ-40 and Aβ-42 were purchased from Thermo Fisher Scientific (Waltham, MA, USA). Dextran was purchased from MilliporeSigma (St. Louis, MO, USA).

### Animals

#### 5xFAD mice

The 5xFAD mice are APP/PS1 double transgenic mice that co-express five FAD mutations and were purchased from the Jackson Laboratory (Bar Harbor, ME, USA). These mice are hemizygous for the amyloid precursor protein (APP) and presenilin 1 (PSEN1) transgenes on a congenic C57BL/6 background and display elevated levels of cerebral Aβ and accelerated Aβ plaque development in the brain, representing some of the pathological features of AD [36].

#### EFAD mice

ApoE-TR mice were purchased from Taconic Biosciences (Rensselaer, NY). The apoE-TR mice were created by targeted replacement of the endogenous murine APOE gene with human APOE2, APOE3 or APOE4 [37] {Sullivan, 1997 #285}. These mice retain the endogenous regulatory sequences required for apoE production and express the human apoE protein at physiological levels. The EFAD animals were provided by Dr. Mary Jo LaDu (University of Illinois at Chicago). To generate the EFAD mouse model, 5xFAD mice (Tg6799 line) were crossed with apoE4, apoE3, and apoE2-TR mice, producing the E4FAD, E3FAD, and E2FAD mouse models respectively, as previously described [38].

#### MMP9KO mice

MMP9KO mice and their C57BL/6 controls were purchased from the Jackson Laboratory (Bar Harbor, ME, USA) and allowed to adapt to the vivarium for 2 weeks prior to any breeding or experimental procedures. MMP9KO mice were generated as previously described and are viable, fertile and shown to survive for at least 24 months [39].

#### Generation of the 5xFAD/MMP9KO mice

The genetic manipulation of MMP9 levels was investigated by crossing MMP9KO mice with 5xFAD mice (both strains were on a C57BL/6 background). 5xFAD mice, hemizygous for the APP and PSEN1 transgenes were initially crossed with MMP9KO mice which were null for the MMP9 gene. The resulting litters were all heterozygous for the MMP9 gene, half were positive for the 5xFAD mutations (5xFAD/MMP9KO-het) and half were negative (wild type (WT)/MMP9KO-het). The 5xFAD/MMP9KO-het mice were then backcrossed with the MMP9KO mice to generate 5xFAD/MMP9KO, WT/MMP9KO (MMP9KO), 5xFAD/MMP9KO-het and WT/MMP9KO-het mice. Concurrent breeding of 5xFAD and WT mice generated the cohort of 5xFAD and WT control mice used for the study. Gender matched, 6-month-old WT, 5xFAD, 5xFAD/MMP9KO, 5xFAD/MMP9KO-het and MMP9KO mice were used in this study (n = 12, 6 males, 6 females). 5xFAD/MMP9KO-het mice were only used for pathological analyses.

#### Housing

Mice were housed under standard laboratory conditions (23 ± 1 °C, 50 ± 5% humidity, and a 12-h light/dark cycle) with free access to food and water throughout the study. Mice were multi-housed through the elevated plus maze (EPM), open field test (OFT) and three-chamber tests. All experiments using animals were performed under protocols approved by the Institutional Animal Care and Use Committee of the Roskamp Institute.

### SB-3CT treatment paradigm and timeline for behavioral analyses

For the pharmacological treatment studies, 4-month-old E4FAD mice were injected intraperitoneally with either SB-3CT (25 mg/kg) dissolved in 25% dimethyl sulfoxide (DMSO)/65% Polyethylene glycol (PEG)-40/10% water or vehicle (25% DMSO/65% PEG-40/10% water alone). It was previously reported that seven days of treatment with this concentration of SB-3CT effectively inhibited MMP9 in a mouse model of focal cerebral ischemia [31]. SB-3CT was designed as a highly selective inhibitor to MMP-2 and MMP-9 with a Ki value of 2.1 nM for MMP-9. Moreover, SB-3CT has a reported brain to plasma AUC ratio of 0.68 and readily distributes throughout the brain, indicating SB-3CT adequately permeates the BBB [35]. The reported elimination half-life of SB-3CT in mice after repeated doses was 53 min [32], using the same dose (25 mg/kg) and route of administration (intraperitoneal) as the current studies. In total, 28 animals were treated, divided into two groups balanced for gender and weight (E4FAD-vehicle, n = 14 (5 × males, 9 × females), E4FAD-SB-3CT, n = 14 (5 × males, 9 × females)). Male and female mice weighed on average 32.41 ± 0.97 g and 21.57 ± 0.21 g, respectively.

### Study design

**Fig. 1:**
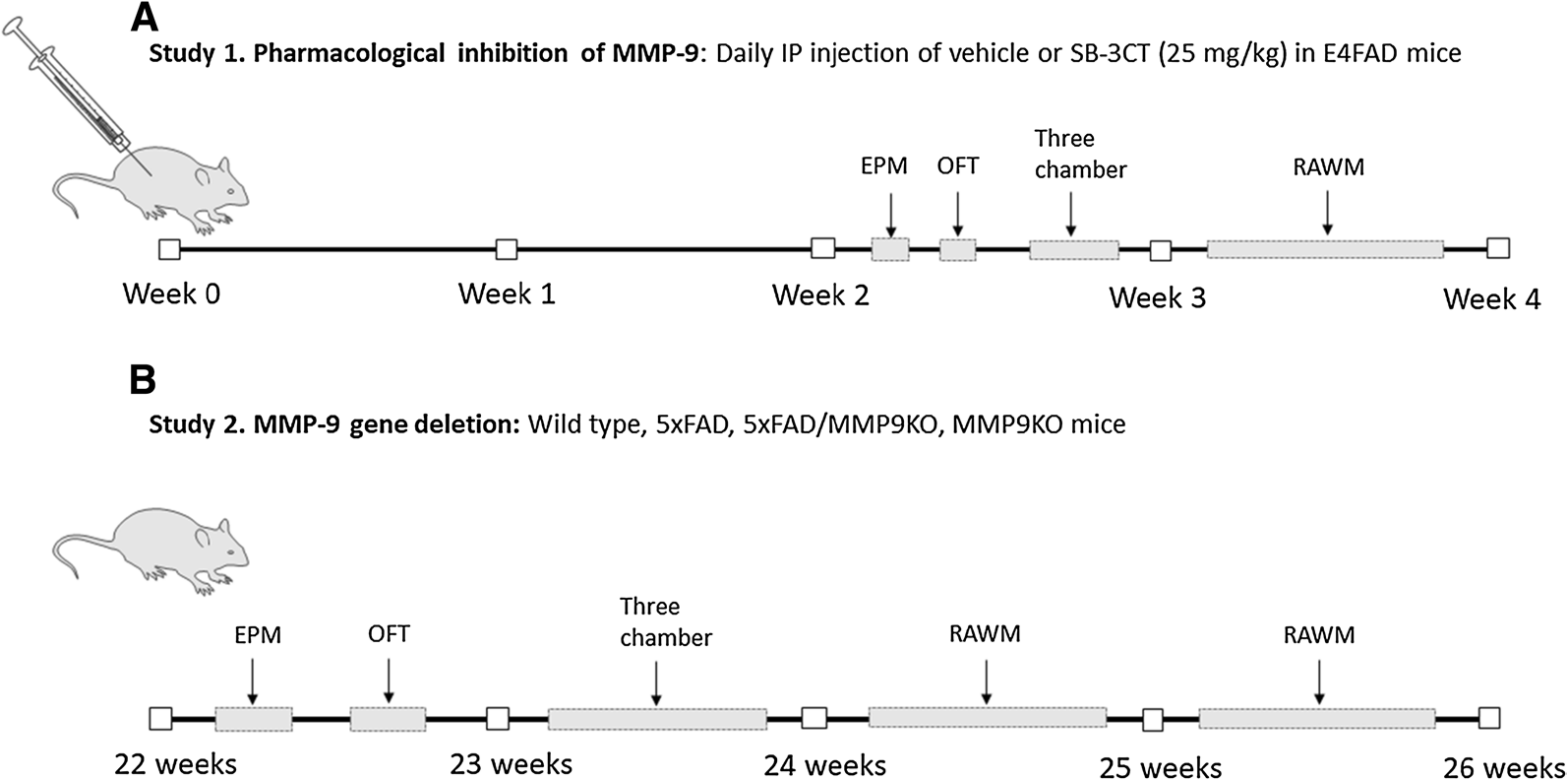
Study design for the 4-week pharmacological MMP9 inhibition and MMP9 gene deletion in vivo analysis. **A** Study 1. MMP9 was pharmacologically inhibited in E4FAD mice through a 4-week treatment with vehicle or SB-3CT (25 mg/kg), injected intraperitoneally (IP). **B** Study 2. MMP9KO mice were crossed with 5xFAD mice to create 5xFAD/MMP9KO mice, which were tested alongside WT, 5xFAD and MMP9KO mice. Behavioral analysis consisted of the elevated plus maze (EPM), open field test (OFT), three chamber test and the radial arm water maze (RAWM). *MMP* Matrix metallopeptidase, *E4FAD* Apolipoprotein E4 x Familial Alzheimer’s disease, *MMP9KO* MMP9 knockout, *WT* Wild-type

Daily intraperitoneal injections (approximately 5PM each day) of vehicle or SB-3CT (25 mg/kg) were administered to 4-month-old E4FAD mice for a period of 4 weeks (Fig. 1a), for a total of 22 injections. Behavioural analysis began 2 weeks after the first injection and mice were euthanized 24 h after the last injection of the 4 week treatment paradigm. For the genetic MMP9 deletion studies, behavioural analyses were performed in WT, 5xFAD, 5xFAD/MMP9KO and MMP9KO mice at 22 weeks of age and mice were euthanized at 26 weeks of age (Fig. 1b), alongside the 5xFAD/MMP9KO-het mice which were used only for the pathological analyses. Euthanasia was performed using 5% isoflurane in oxygen via inhalation using a vaporizer at 1 L/min, followed by decapitation.

### Behavioral analysis

#### Evaluation of anxiety-related behavior and motor activity in mice

Motor function and anxiety were assessed in E4FAD mice after 2 weeks of treatment with either SB-3CT (25 mg/kg) or vehicle using the elevated plus maze and the open field test. These same tests were utilized in the MMP9 gene deletion studies to evaluate the WT, 5xFAD, 5xFAD/MMP9KO and MMP9KO animals. The EPM consists of an elevated area (0.5 m) with two open arms and two closed arms with 15 cm high walls and an open roof (similar arms are opposite each other) [40]. Mice were individually placed in the center of the maze and movements were tracked using the EthoVision software for 5 min (Noldus, VA, USA). Mice were scored based on the number of entries into closed vs open arms and the time spent in closed vs open arms. An increase in open arm activity indicates anti-anxiety behavior [41]. The OFT is a common measure of exploratory behavior and general activity in mice [42–44]. The mice were individually placed into an enclosure with surrounding walls and an open roof and movements were tracked using the EthoVision software for 10 min (Noldus, VA, USA). Mice were scored based on the number of entries into the center, middle and outer edges of the arena and the time spent in these three areas. An increase in duration/number of entries into the center area indicates anti-anxiety behavior [42–44]. The distanced travelled by the animal in the OFT provides a measure of motor activity [42, 43].

#### Assessment of social interaction and social memory in mice

The three-chamber test was used to measure cognition in the form of general sociability and interest in social novelty [45] in the E4FAD mice following 2.5 weeks of SB-3CT treatment. Additionally this test was used in the MMP9 gene deletion studies to evaluate the WT, 5xFAD, 5xFAD/MMP9KO and MMP9KO animals. In this test, mice were placed individually into the center chamber of a box arena with three equally sized chambers and openings between the chambers. A schematic of the setup is displayed in Figs. 3 and 10. The two side chambers contained a wire cup through which the subject mouse can have indirect interaction with the novel and familiar mice. This test consisted of three 10-min experimental sessions with different setups. In all sessions, the subject mouse could explore the whole arena. In the first session (habituation) the two side chambers remained empty to give the mice time to explore and become familiar with the arena. In the second session (test for social interaction), a mouse was added to the wire cup in one side chamber. In the third session (test for social memory), a novel mouse was placed in the wire cup in the opposite side chamber to the now familiar mouse. Positions of the novel and familiar mice were changed between trials to avoid side bias. Time spent in each chamber, time spent in the immediate area surrounding the wire cup, and the number of entries into each area were recorded. The test for social interaction measured the time spent with another mouse compared to time spent alone in an identical but empty chamber. The test for social memory measured the preference for a novel vs familiar mouse [45].

#### Assessment of spatial memory in mice

Spatial memory and learning was assessed in E4FAD mice after 3 weeks of treatment with either vehicle or SB-3CT using the radial arm water maze (RAWM) [46]. This test was also used in the MMP9 gene deletion studies evaluating the WT, 5xFAD, 5xFAD/MMP9KO and MMP9KO animals. The RAWM consists of a circular water-filled maze with six arms extending from an open central area that are distinguishable by unique visual cues on the end of each arm. A schematic of the setup is displayed in Figs. 4 and 12. The mice were placed into one of the five entry arms (alternated between trials) and the task was to find the hidden platform in the sixth arm (goal arm), which remained constant. Each trial ended after one minute and errors were calculated after tracking the movements using the EthoVision software (Noldus, VA, USA). Entry into an incorrect arm was scored as an error. The total number of incorrect errors made per trial before finding the hidden platform reflects reference memory, while the number of incorrect re-entries (multiple entries into the same arm) indicates working memory. The mice underwent nine trials per day for a total of five consecutive days. By the last day of trials, mice that have correctly learned the location of the hidden platform demonstrate errors of 1 or less and show improvement between trials [46]. Latency to reach the hidden platform was also recorded and analyzed.

#### Isolation of brain fractions

Mouse brains were homogenized and the cerebrovasculature, parenchyma and soluble brain fraction were isolated using a step-wise density gradient extraction process as previously described [16]. Briefly, mouse brain samples were homogenized in cold HBSS using a Dounce homogenizer. The homogenates were suspended in HBSS with 20% dextran and centrifuged for 15 min at 6000 g and 4 °C. The cerebrovascular pellet at the bottom of the tube was gently rinsed in HBSS and collected with lysis buffer (mammalian protein extraction (M-PER) reagent + 1% ethylenediaminetetraacetic acid (EDTA) + 0.2% phenylmethylsulphonyl fluoride (PMSF) (Thermo Scientific, USA)) supplemented with Halt protease and phosphatase inhibitor cocktail (Thermo Scientific, USA). The remaining parenchyma and soluble brain fraction (i.e., non-cell associated) were centrifuged for a further 10 min to separate these two fractions. The parenchyma was resuspended in HBSS and centrifuged for a final 5 min before the pellet was collected in lysis buffer. All fractions were stored at − 80 °C prior to analysis. Quantification of Aβ40 and Aβ42 in the whole parenchyma, cerebrovascular and plasma fractions was carried out using an ELISA for human Aβ40 and Aβ42 (Invitrogen, USA).

#### Zymographic analysis of EFAD spleen samples

Spleen samples from SB-3CT and placebo-treated E4FAD mice were analyzed for MMP9 content through zymographic analysis. Lysis buffer (M-PER + 1% EDTA + 0.2% PMSF (Thermo Scientific, USA)) supplemented with Halt protease and phosphatase inhibitor cocktail (Thermo Scientific, USA) was added to spleen samples collected from EFAD mice before they were homogenized via sonication (Sonic Dismembrator model 100, T). Samples were centrifuged to remove cell debris before analysis by gelatin zymography to determine pro and active MMP9 levels. Equal protein quantities of each sample (50 µg) were incubated with Gelatin-Sepharose® 4B (GE Healthcare, Chicago IL) to concentrate the MMP9. Samples were incubated with the beads for 1–2 h at room temperature with rotation, and then centrifuged at 6000 rpm for 2 min. Gelatinases were eluted in equal amounts (25 µL) of 1X Zymogram sample buffer (Bio-Rad, Hercules, CA, USA) before being separated on a 10% precast polyacrylamide gel with gelatin (Thermo Fisher Scientific, Waltham, MA, USA). The gel was incubated in Triton X-100 (Zymogram Renaturation Buffer, Thermo Fisher Scientific, Waltham, MA, USA) for 30 min at room temperature with gentle agitation to renature the proteins. The gel was next incubated in development buffer containing 50 mM Tris–HCl, pH 7.5, 200 mM NaCl, 5 mM CaCl2, 0.02% Brij-35 (Zymogram Development Buffer, Thermo Fisher Scientific, Waltham, MA, USA) for 18 h at 37 °C to initiate enzyme activity. The gel was stained with 0.5% Coomassie blue (Bio-Rad, Hercules, CA, USA) for one hour and washed in destaining solution (45% deionised water, 45% methanol, 10% acetic acid) before being scanned with the Universal Hood II (Bio-Rad, Hercules, CA, USA).

#### Statistical analysis

Data are expressed as mean ± standard error of the mean (SEM). Data was checked for normality and statistical significance was determined by analysis of variance (ANOVA) followed by the two-stage step-up method of Benjamini, Krieger and Yekutieli (BKY) unless otherwise stated. A p-value lower than 0.05 was used to indicate a statistically significant difference. Statistical analyses were performed with GraphPad Prism 8.

## Results

### Pharmacological inhibition of MMP9 activity with SB-3CT in E4FAD mice

#### SB-3CT treatment influenced anxiety levels but not motor activity in E4FAD mice

**Fig. 2:**
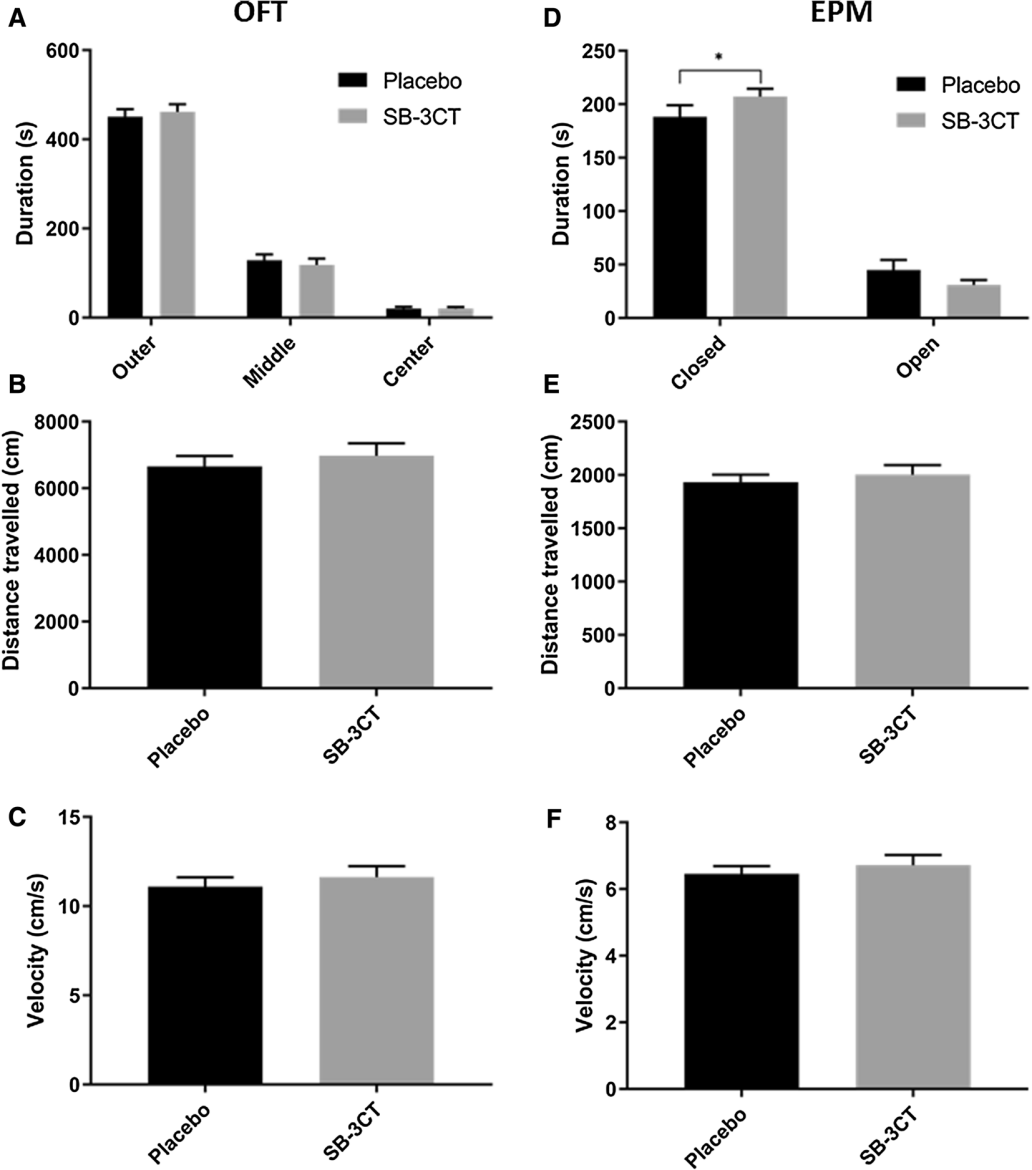
Anxiety-related behavior and locomotor activity in the OFT and the EPM. SB-3CT and vehicle-treated E4FAD mice were tested using **A**, **B**, **C** the OFT and **D**, **E**, **F**, the EPM. **A** For the OFT, the duration spent in the outer, middle and center areas of the circular arena was measured along with B the total distance travelled and **C** the average velocity. **D** For the EPM, the duration spent in the closed and open arms was measured together with **E** the total distance travelled and **F** the average velocity. Animals were approximately 5 months of age and values represent mean ± SEM (N = 14, 5 males, 9 females for each group). Statistical significance was determined by **A**, **D** ANOVA followed by the BKY procedure and **B**, **C**, **E**, **F** an unpaired t-test. *p < 0.05. *EPM* Elevated plus maze, *OFT* Open field test, *E4FAD* Apolipoprotein E4 x Familial Alzheimer’s disease, *SEM* Standard error of the mean, *ANOVA* Analysis of variance, *BKY* Benjamini, Krieger, and Yekutieli

Following 2 weeks of daily SB-3CT injections, the EPM and the OFT revealed no differences in total distance travelled or average velocity between the SB-3CT-treated and vehicle-treated mice (Fig. 2b, c, e, f), indicating the drug treatment does not alter locomotor activity. Furthermore, while no effects in anxiety were demonstrated in the OFT (Fig. 2a), SB-3CT-treated mice spent significantly more time in the closed arm compared to control animals in the EPM (*p < 0.05) (Fig. 2d). There were no gender differences observed in either the EPM or the OFT.

#### SB-3CT treatment did not impact social interaction, social memory, or spatial memory

**Fig. 3:**
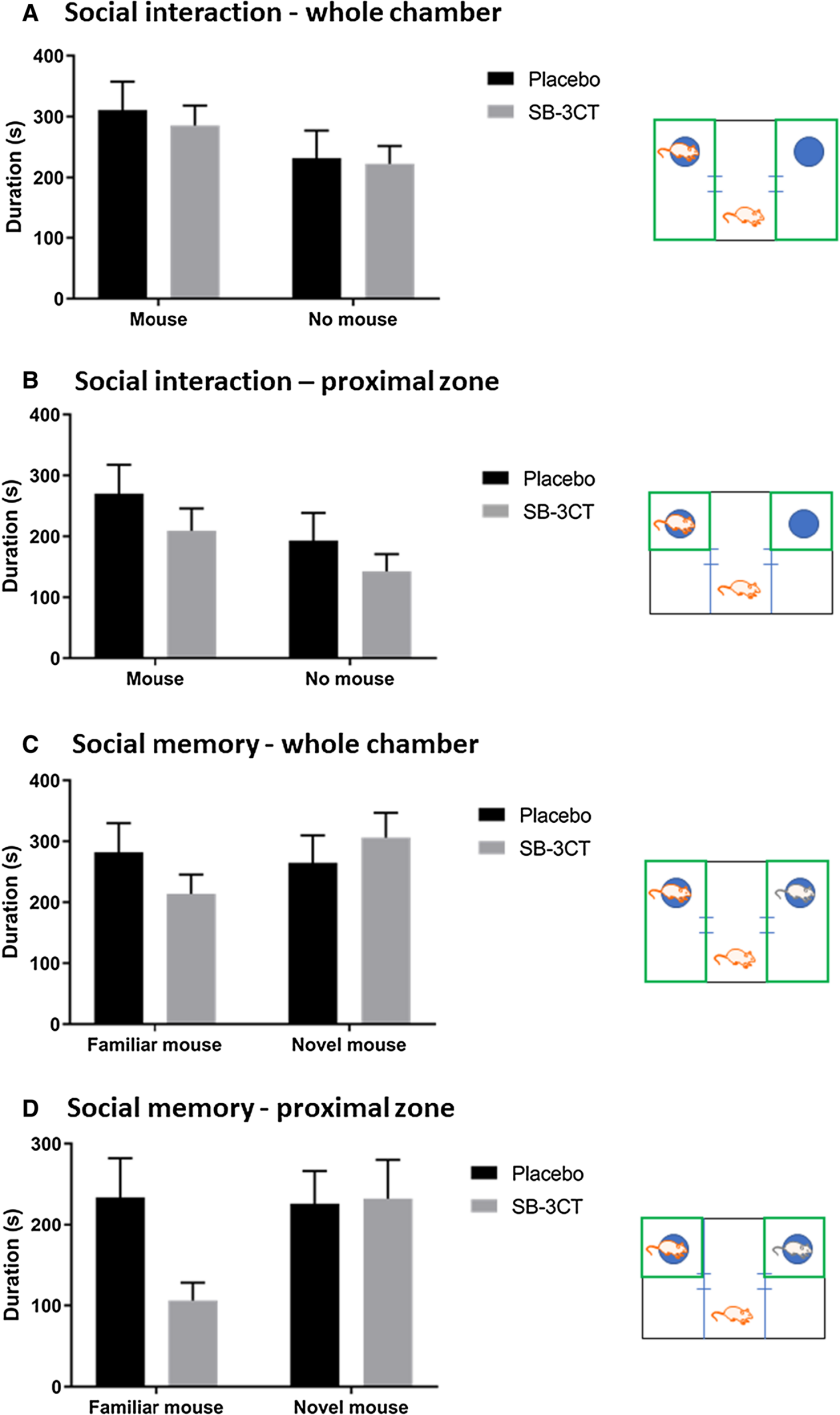
Testing social interaction and social memory using the three-chamber test. SB-3CT and vehicle-treated E4FAD mice were tested for **A**, **B** social interaction (one mouse vs empty cage) and **C**, **D** social memory (novel mouse vs familiar mouse) in the three-chamber test. Time spent in **A**, **C** the whole chamber containing the mouse/empty cage and **B**, **D** the proximal zone surrounding the cages was measured (areas shown in green in each accompanying schematic). Animals were approximately 5 months of age and values represent mean ± SEM (N = 14, 5 males, 9 females for each group). No statistical significance was identified in any measures by ANOVA. *E4FAD* Apolipoprotein E4 x Familial Alzheimer’s disease, *SEM* Standard error of the mean, *ANOVA* Analysis of variance

**Fig. 4:**
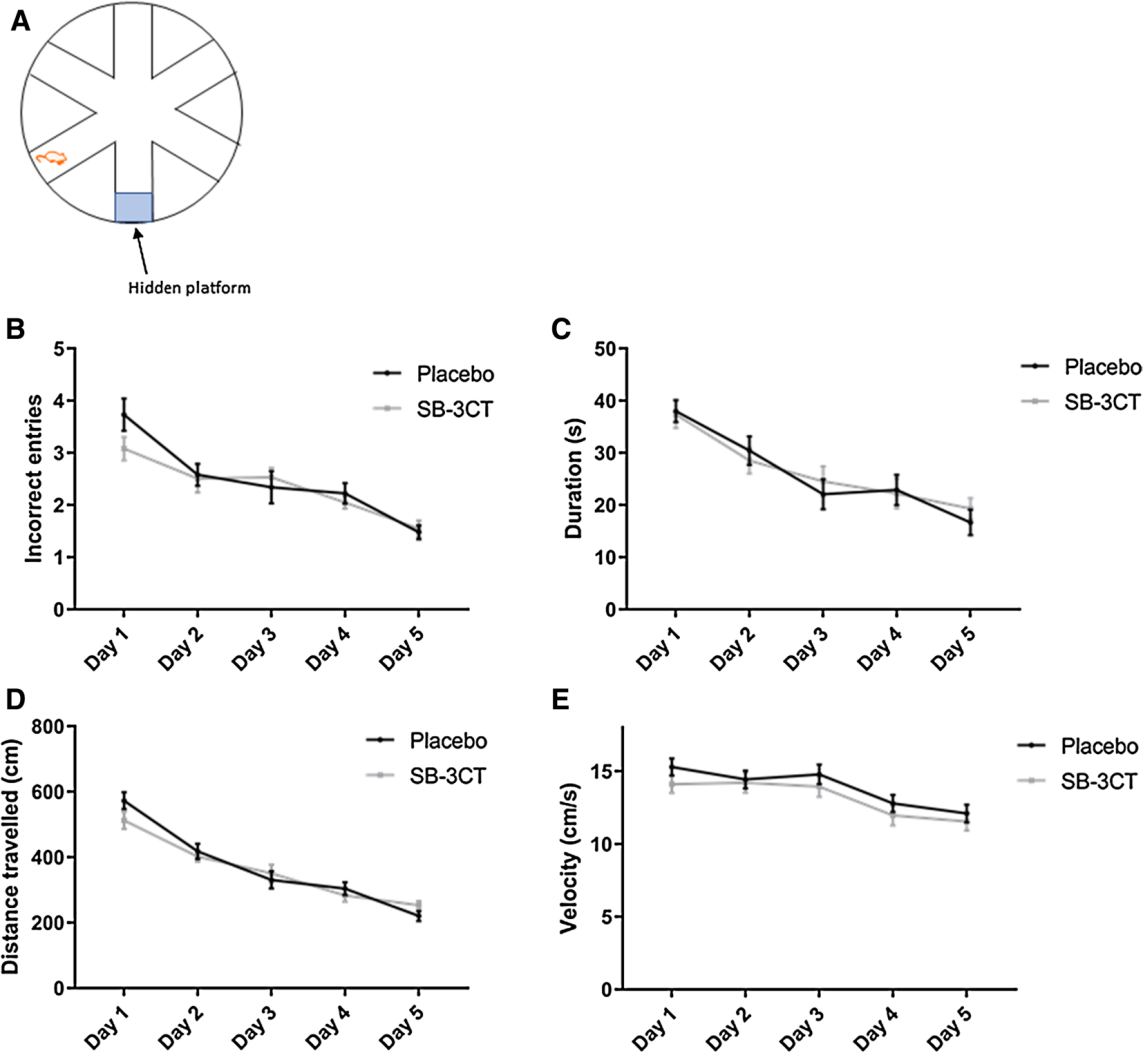
Spatial memory testing using the RAWM. SB-3CT and vehicle-treated E4FAD mice were tested for their ability to find the hidden platform in the RAWM. **A** A schematic of the maze layout is displayed. Mice were tested in nine trials per day for 5 days. **B** The number of incorrect entries made and **C** the time taken to find the maze were recorded and analyzed. **D** The total distance travelled per trial and **E** the average velocity while swimming was also evaluated. Animals were approximately 5 months of age and values represent mean ± SEM (N = 14, 5 males, 9 females for each group). No statistical significance was identified in any of the parameters by ANOVA. *RAWM* Radial arm water maze, *E4FAD* Apolipoprotein E4 x Familial Alzheimer’s disease, *SEM* Standard error of the mean, *ANOVA* Analysis of variance

**Fig. 5:**
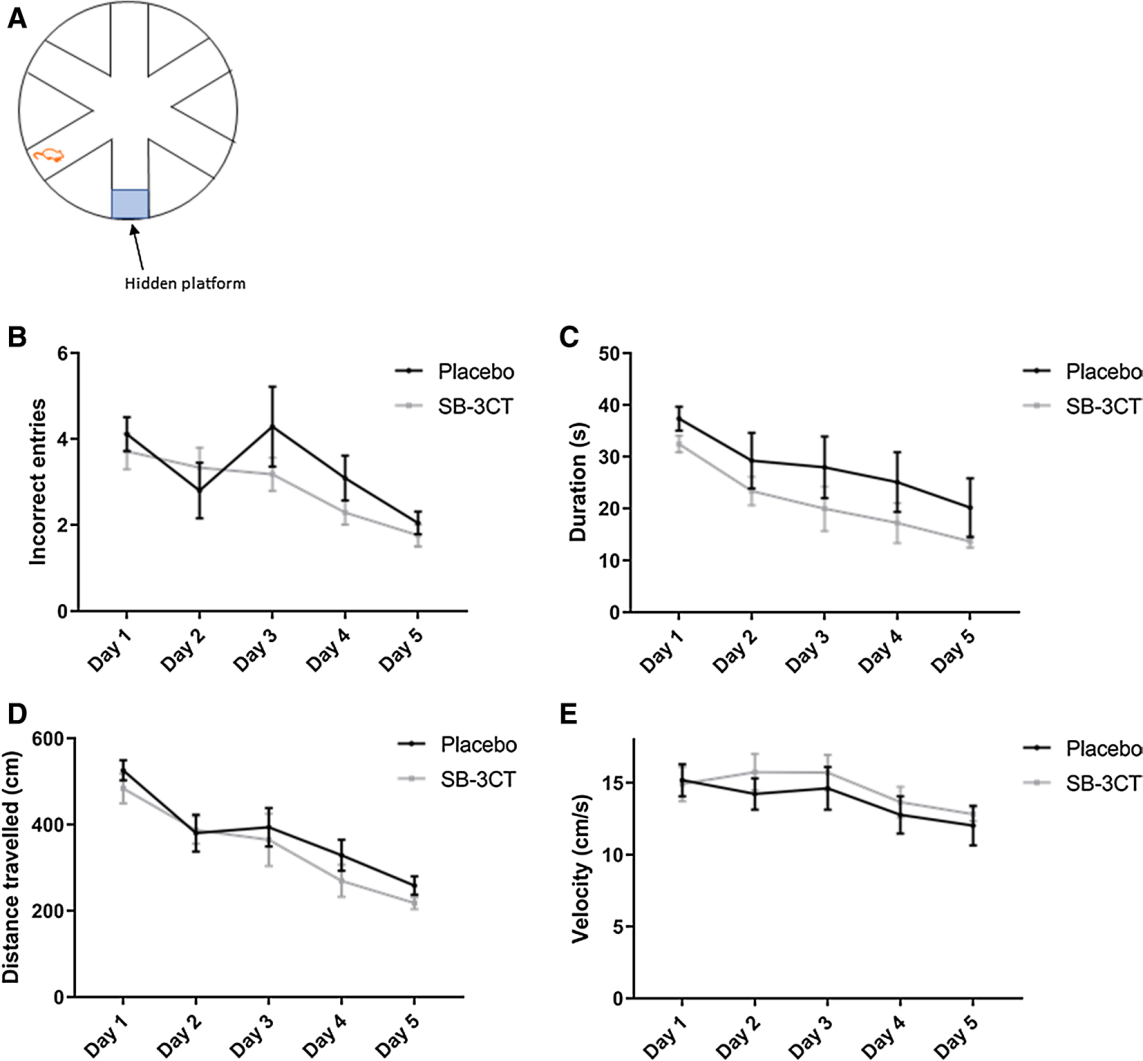
Spatial memory testing of male mice using the RAWM. SB-3CT and vehicle-treated male E4FAD mice were tested for their ability to find the hidden platform in the RAWM. **A** A schematic of the maze layout is displayed. Mice were tested in nine trials per day for 5 days. **B** The number of incorrect entries made and **C** the time taken to find the maze were recorded and analyzed. **D** The total distance travelled per trial and **E** the average velocity while swimming was also evaluated. Values represent mean ± SEM (N = 5 males for each group). No statistical significance was identified in any of the parameters by ANOVA. *RAWM* Radial arm water maze, *E4FAD* Apolipoprotein E4 x Familial Alzheimer’s disease, *SEM* Standard error of the mean, *ANOVA* Analysis of variance

E4FAD mice treated with either SB-3CT or vehicle showed no preference for the chamber or proximal zone containing a mouse compared to an empty cage in the three-chamber test (Fig. 3a, b). Furthermore, there were no differences between either group in the time spent with a novel mouse compared to a familiar mouse (Fig. 3c, d). Moreover, there were no gender differences observed in the three-chamber test. In the RAWM, no differences between the SB-3CT or vehicle-treated mice were observed in the number of incorrect entries made before finding the hidden platform (Fig. 4b), the latency to find the platform (Fig. 4c), the average distance travelled per trial (Fig. 4d) or the average velocity (Fig. 4e). Overall mice in both treatment groups continued to learn and improve each of the 5 days, making few incorrect entries and finding the hidden platform relatively quickly. When stratified for gender (Fig. 5a, b, c, d, e), male mice treated with SB-3CT displayed a slightly reduced latency to find the hidden platform, however this was not statistically significant (Fig. 5c).

#### Brain Amyloid levels were similar in SB-3CT and vehicle-treated E4FAD mice

**Fig. 6:**
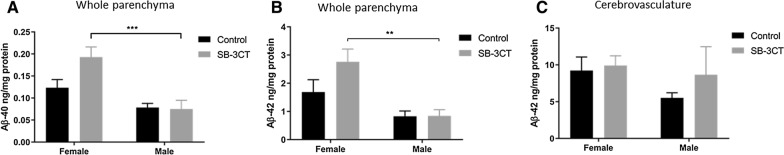
Analysis of Aβ-40 and Aβ-42 levels in the whole parenchyma and cerebrovascular brain fractions. Aβ-40 levels were examined in the **A** whole parenchyma, while Aβ-42 levels were examined in the **B** whole parenchyma and **C** cerebrovasculature of E4FAD mice. Males and females were analyzed separately due to large differences in amyloid levels. Animals were approximately 5 months of age and values represent mean ± SEM (N = 14, 5 males, 9 females for each group). Statistical significance was determined by ANOVA followed by the BKY procedure. *p < 0.05, **p < 0.01 ***p < 0.001. *Aβ* Beta-amyloid, *E4FAD* Apolipoprotein E4 x Familial Alzheimer’s disease, *SEM* Standard error of the mean, *ANOVA* Analysis of variance, *BKY* Benjamini, Krieger, and Yekutieli

Aβ-40 and Aβ-42 levels were evaluated in the brain parenchyma fractions of SB-3CT and vehicle-treated E4FAD mice. The whole parenchyma brain fraction was analyzed in addition to Aβ-42 levels in the cerebrovasculature of each mouse. While no differences in Aβ-40 and Aβ-42 levels were observed in any brain fraction when comparing SB-3CT to vehicle-treated animals, Aβ-40 and Aβ-42 levels were higher in female mice compared to male mice in the whole parenchyma brain fraction (*p < 0.05, **p < 0.01, ***p < 0.001) (Fig. 6).

#### LDLR and LRP1 levels were unchanged across treatment groups in E4FAD mice

**Fig. 7:**
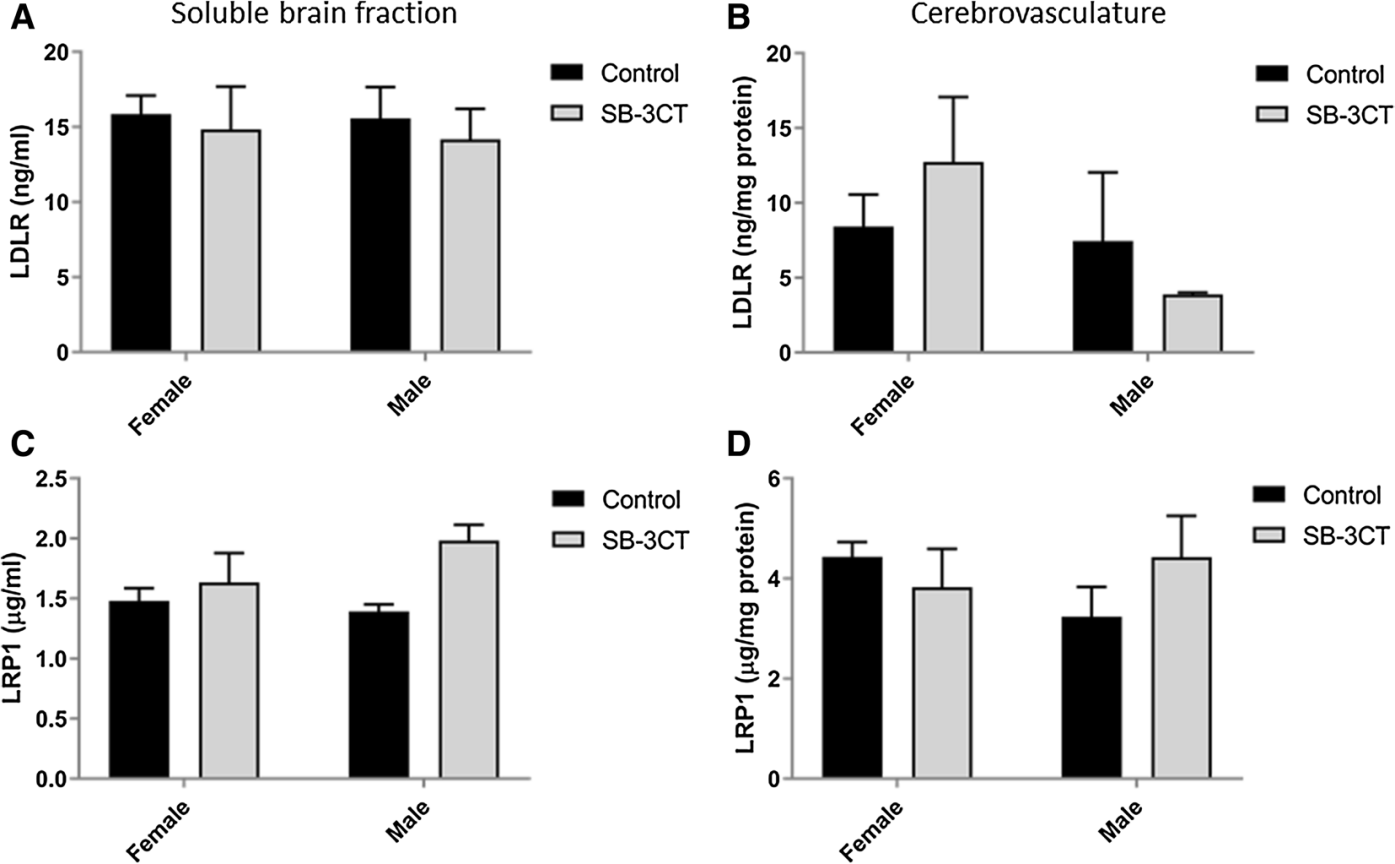
Analysis of LDLR and LRP1 levels in the cerebrovasculature and the soluble brain fraction. Levels of the **A**, **B** LDLR receptor and **C**, **D** LRP1 receptor were analyzed in the **A**, **C** soluble brain fraction and **B**, **D** the cerebrovasculature of SB-3CT and vehicle-treated E4FAD mice. Animals were approximately 5 months of age and values represent mean ± SEM (N = 14, 5 males, 9 females for each group). No statistically significant differences in LDLR or LRP1 levels were identified between vehicle or SB-3CT-treated mice in either brain fraction by ANOVA. *LDLR* Low-density lipoprotein receptor, *LRP1* Low density lipoprotein receptor-related protein 1, *E4FAD* Apolipoprotein E4 x Familial Alzheimer’s disease, *SEM* Standard error of the mean, *ANOVA* Analysis of variance

LDLR and LRP1 were measured in the cerebrovasculature and soluble brain fraction of SB-3CT and vehicle-treated E4FAD mice (Fig. 7). The levels of both receptors were not different between the SB-3CT and vehicle-treated groups for both brain fractions of (Fig. 7a, b, c, d). Moreover, there were no gender differences observed in the analysis of LDLR or LRP1.

#### Pro and total MMP9 levels were unaltered by SB-3CT treatment

**Fig. 8:**
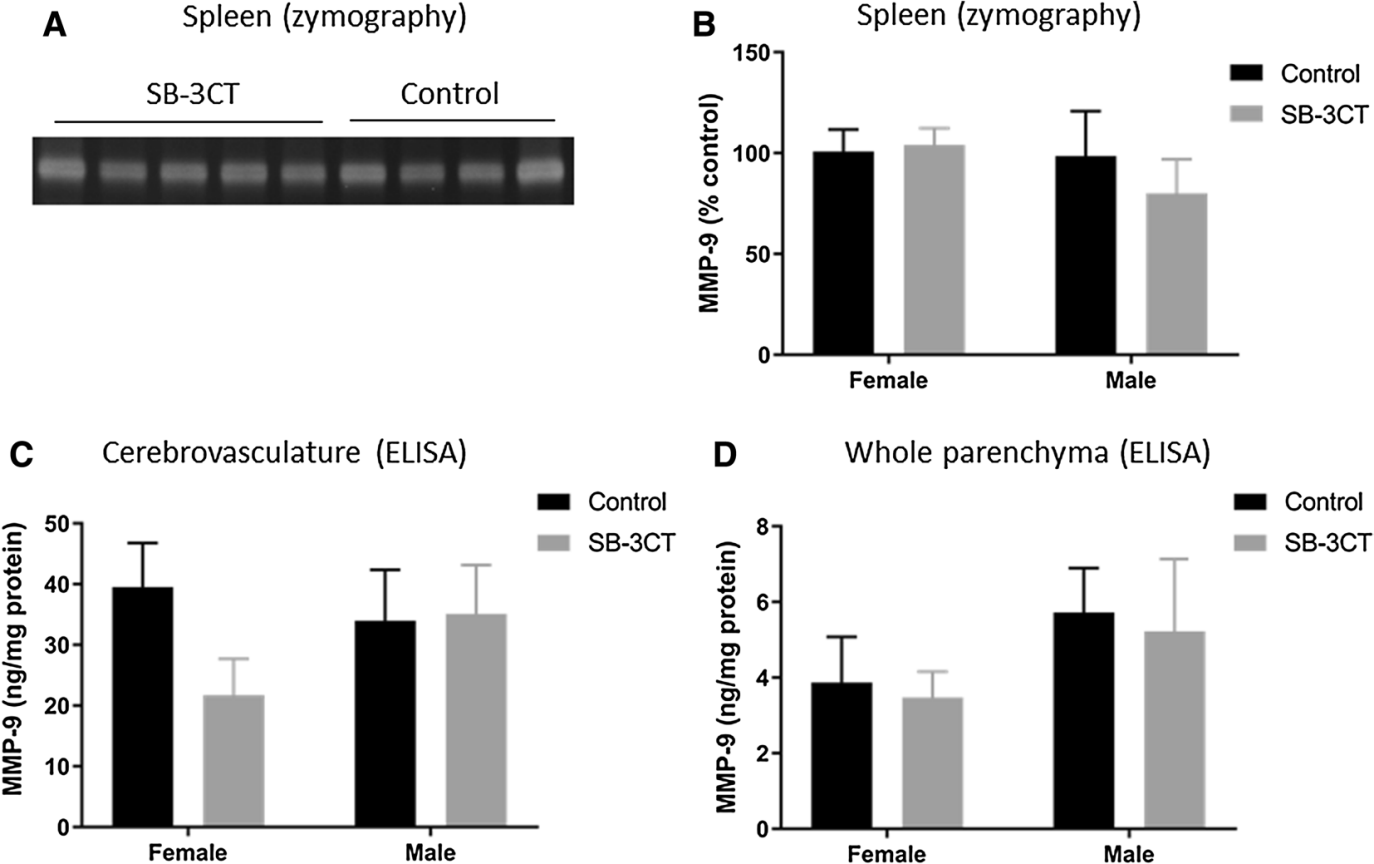
Analysis of MMP9 levels in SB-3CT and vehicle-treated E4FAD mice. **A**, **B** Levels of proMMP9 were examined in spleen samples from SB-3CT and vehicle-treated mice by zymography. **A** Zymography gel showing bands of proMMP9 in SB-3CT-treated and control animals. **B** Quantification of zymographic analysis. **C**, **D** Levels of total MMP9 as measured by ELISA analysis of the **C** cerebrovasculature and **D** the whole brain parenchyma of SB-3CT and vehicle-treated E4FAD mice. Animals were approximately 5 months of age and values represent mean ± SEM (N = 14, 5 males, 9 females for each group). No statistical significance was identified in any fraction by ANOVA. *MMP* Matrix metallopeptidase, *E4FAD* Apolipoprotein E4 x Familial Alzheimer’s disease, *ELISA* Enzyme linked immunosorbent assay, *SEM* Standard error of the mean, *ANOVA* Analysis of variance

To examine the effects of the MMP9 inhibitor (SB-3CT) on MMP9 activity, the spleens of each mouse were examined via zymography (Fig. 8a, b), owing to the high expression levels of MMP9 in this tissue type [47–49]. ProMMP9 levels were detected in the spleens of the mice; however, no differences were identified between mice treated with SB-3CT or vehicle (Fig. 8a, b). Of note, neither pro-MMP9 nor active MMP9 levels were high enough in the mouse brain tissue samples to be detected via zymographic analysis and therefore these analyses were restricted to spleen samples only. To investigate whether total MMP9 expression levels in the brain were altered by SB-3CT treatment, cerebrovasculature and parenchyma brain samples were analyzed for MMP9 using an ELISA. No differences in total MMP9 levels were identified between SB-3CT and vehicle-treated mice in either fraction (Fig. 8c, d). Of note, no side effects or signs of toxicity were observed with this treatment paradigm, consistent with previous reports administering SB-3CT to mice using the same dose and a similar treatment regimen as the current studies [31, 32].

### Genetic manipulation of MMP9 in 5xFAD mice

#### Anxiety and motor function differ between 5 and 5xFAD/MMP9KO mice

**Fig. 9:**
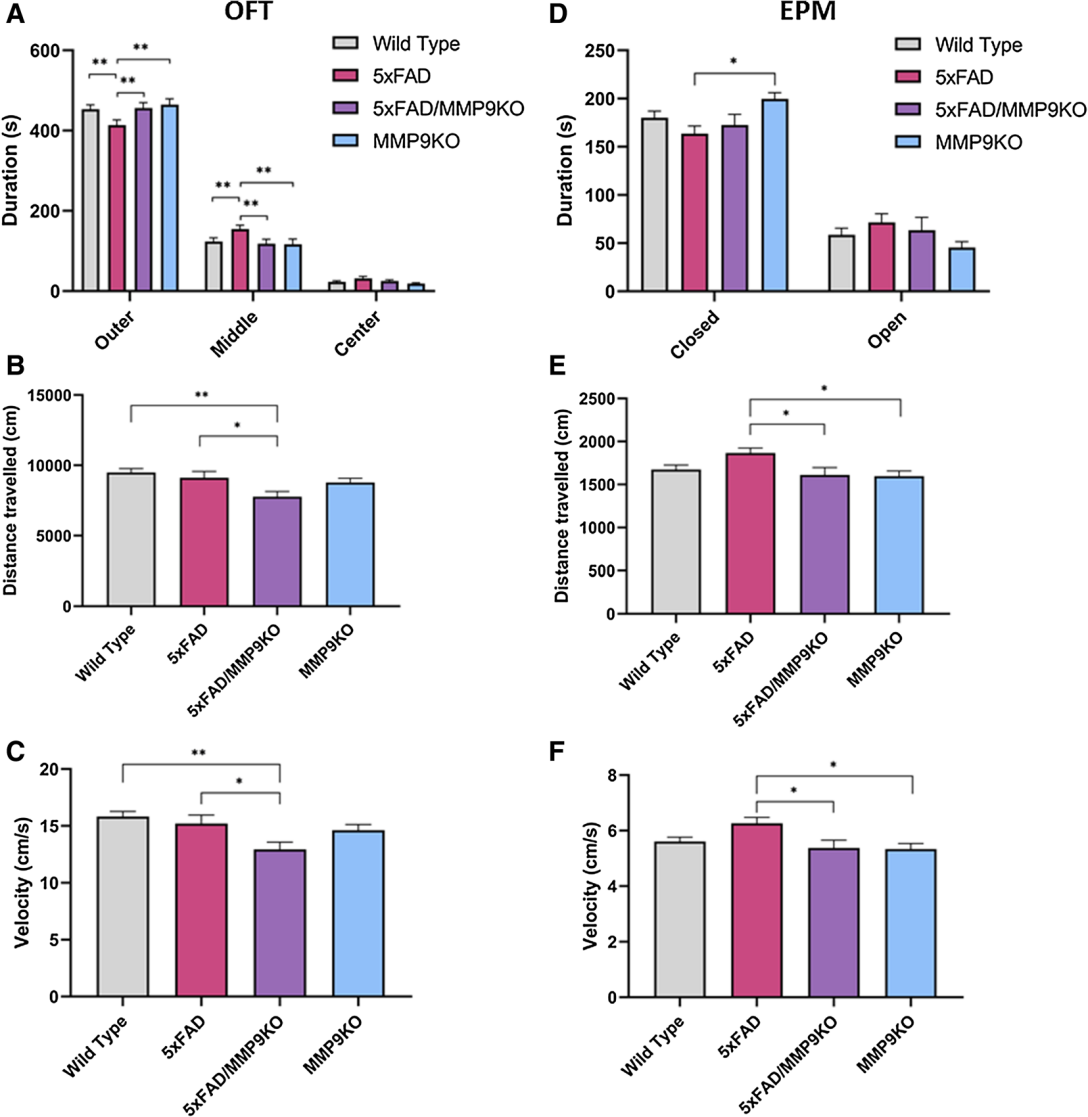
Anxiety-related behavior and locomotor activity in the OFT and the EPM. Wild type, 5xFAD, 5xFAD/MMP9KO and MMP9KO mice were tested using **A**, **B**, **C** the OFT and **D**, **E**, **F**, the EPM. **A** For the OFT, the duration spent in the outer, middle, and center areas of the circular arena was measured along with **B** the total distance travelled and **C** the average velocity. **D** For the EPM, the duration spent in the closed and open arms was measured together with **E** the total distance travelled and **F** the average velocity. Animals were approximately 6 months of age and values represent mean ± SEM (N = 12, 6 males, 6 females for each group). Statistical significance was determined by (**A**, **D**) ANOVA and (**B**, **C**, **E**, **F**) one-way ANOVA followed by the BKY procedure. *p < 0.05, **p < 0.01. *OFT* Open field test, *EPM* Elevated plus maze, *WT* Wild-type, *MMP* Matrix metallopeptidase, *FAD* Familial Alzheimer’s disease, *MMP9KO* MMP9 knockout, *SEM* Standard error of the mean, *ANOVA* Analysis of variance, *BKY* Benjamini, Krieger, and Yekutieli

The OFT demonstrated that 5xFAD mice exhibit reduced anxiety compared to all other genotypes (Fig. 9a). The WT, 5xFAD/MMP9KO, and MMP9KO mice spent more time in proximity to the outer walls of the arena, while 5xFAD mice spent significantly less time in this area (**p < 0.01). Instead, 5xFAD mice spent significantly more time in the middle area of the arena, away from the walls, than the other genotypes (*p < 0.05) (Fig. 9a). For the EPM, results showing the time spent in the closed and open arms indicate the 5xFAD mice spent less time in the closed arms compared to MMP9KO mice (*p < 0.05), but no significant difference was observed between the 5xFAD and WT mice (Fig. 9d). In the OFT, the total distance travelled and the average velocity were both reduced in 5xFAD/MMP9KO compared to 5xFAD and WT mice (*p < 0.05, **p < 0.01) (Fig. 9b, c). In the EPM, these parameters were both increased in 5xFAD mice compared to 5xFAD/MMP9KO and MMP9KO mice (*p < 0.05) (Fig. 9e, f). There were no gender differences observed in either the EPM or the OFT.

#### Genetic deletion of MMP9 rescues social memory deficits in 5xFAD mice

**Fig. 10:**
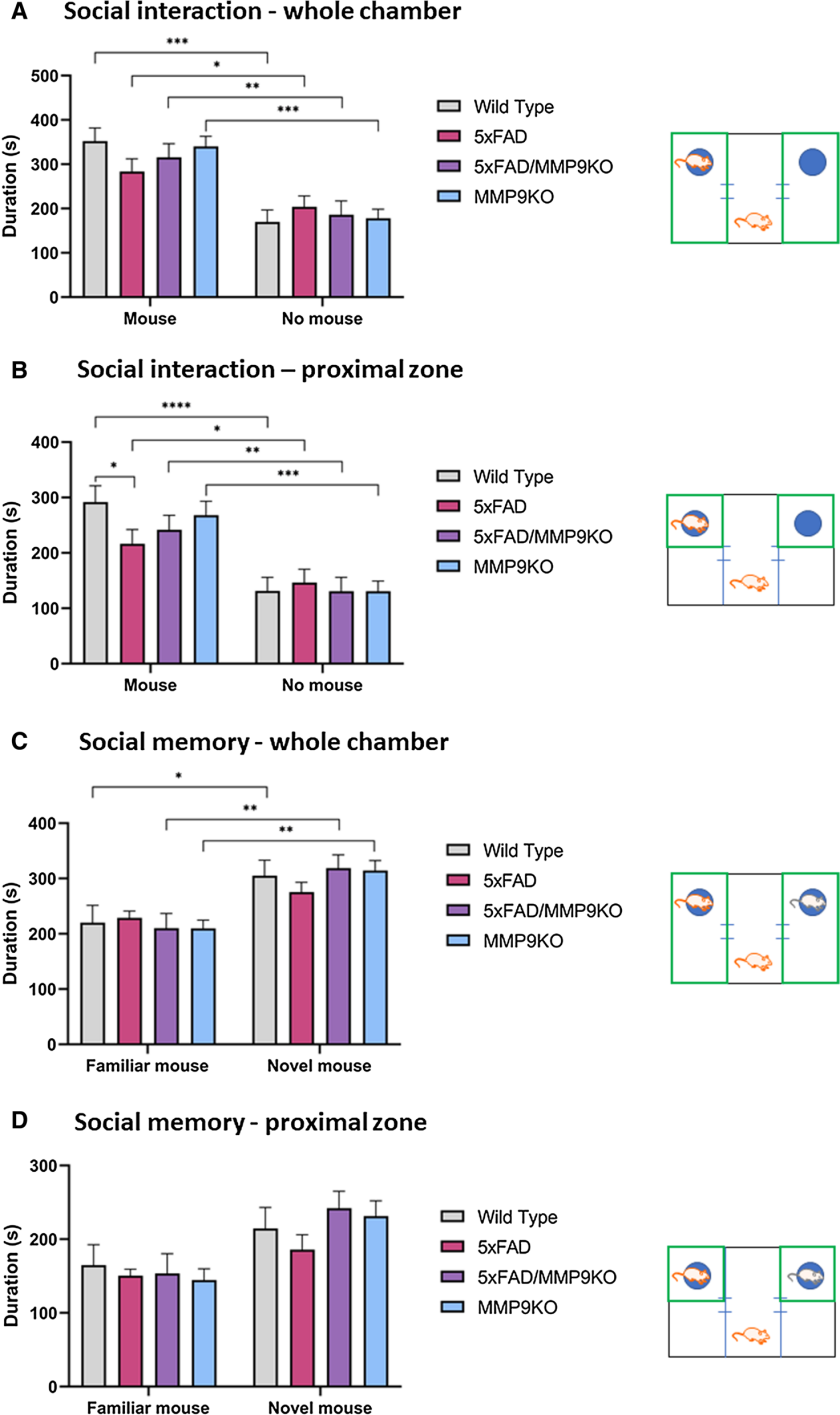
Testing social interaction and social memory using the three-chamber test. Wild type, 5xFAD, 5xFAD/MMP9KO and MMP9KO mice were tested for **A**, **B** social interaction (one mouse vs empty cage) and **C**, **D** social memory (novel mouse vs familiar mouse) in the three-chamber test. Time spent in **A**, **C** the whole chamber containing the mouse/empty cage and **B**, **D** the proximal zone surrounding the cages was measured (areas shown in green in each accompanying schematic). Animals were approximately 6 months of age and values represent mean ± SEM (N = 12, 6 males, 6 females for each group). Statistical significance was determined by ANOVA followed by the BKY procedure. *p < 0.05, **p < 0.01, ***p < 0.001, ****p < 0.0001. *WT* Wild-type, *MMP* Matrix metallopeptidase, *FAD* Familial Alzheimer’s disease, *MMP9KO* MMP9 knockout, *SEM* Standard error of the mean, *ANOVA* Analysis of variance, *BKY* Benjamini, Krieger, and Yekutieli

**Fig. 11:**
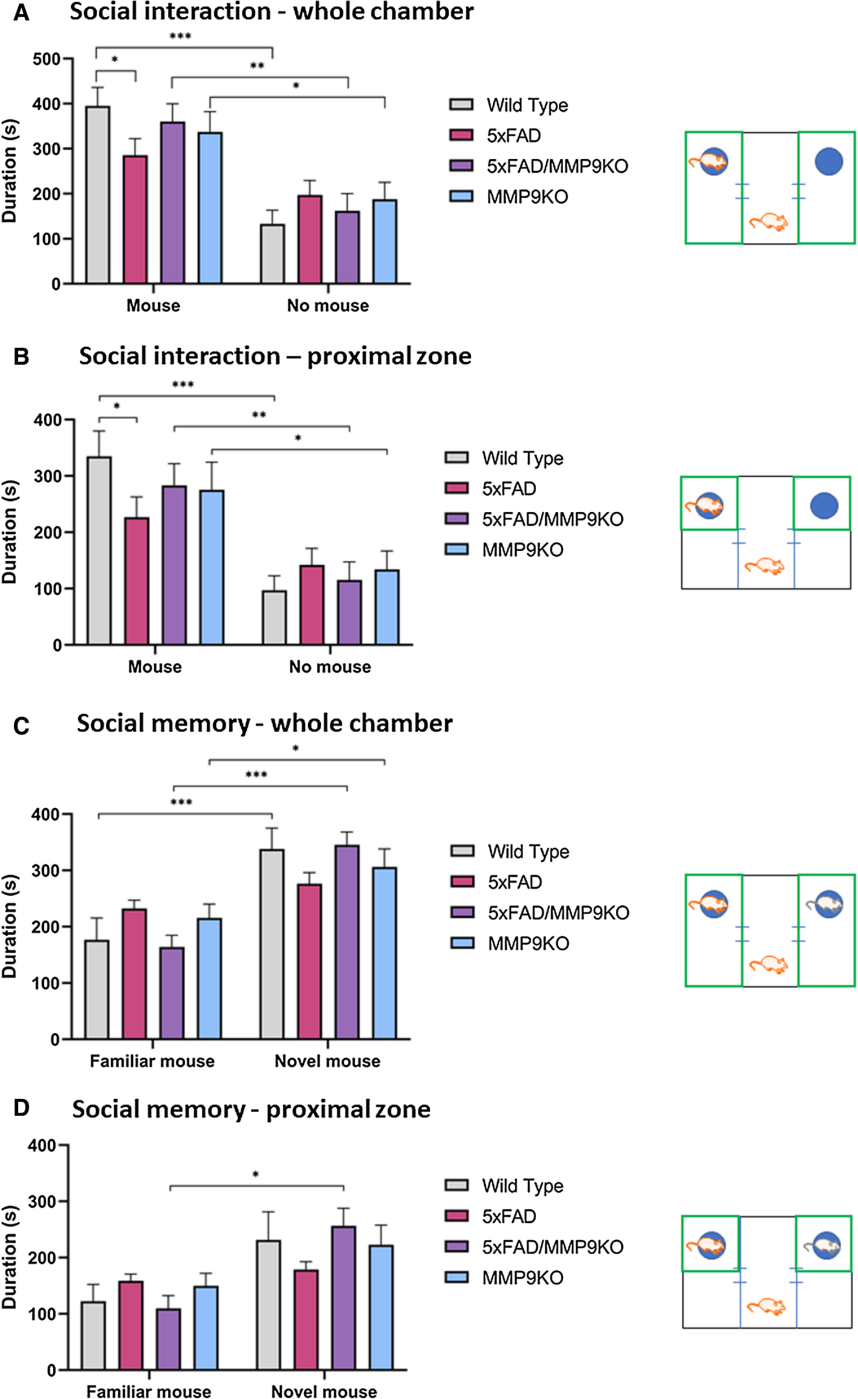
Testing social interaction and social memory of male mice using the three-chamber test. Male wild type, 5xFAD, 5xFAD/MMP9KO and MMP9KO mice were tested for **A**, **B** social interaction (one mouse vs empty cage) and **C**, **D** social memory (novel mouse vs familiar mouse) in the three-chamber test. Time spent in **A**, **C** the whole chamber containing the mouse/empty cage and **B**, **D** the proximal zone surrounding the cages was measured (areas shown in green in each accompanying schematic). Animals were approximately 6 months of age and values represent mean ± SEM (N = 6 males for each group). Statistical significance was determined by ANOVA followed by the BKY procedure. *p < 0.05, **p < 0.01, ***p < 0.001. *WT* Wild-type, *MMP* Matrix metallopeptidase, *FAD* Familial Alzheimer’s disease, *MMP9KO* MMP9 knockout, *SEM* Standard error of the mean, *ANOVA* Analysis of variance, *BKY* Benjamini, Krieger, and Yekutieli

The EPM and OFT were followed by the three-chamber test to assess social interaction behavior (general sociability) and interest in social novelty. Regarding sociability, all genotypes spent significantly more time in the chamber with another mouse compared to the chamber with an empty cage (*p < 0.05, **p < 0.01, ***p < 0.001) (Fig. 10a). When examining the time spent in close proximity to the empty cage or the cage containing a mouse, again, all genotypes spent more time in the proximal zone surrounding the cage containing the mouse compared to the empty cage (*p < 0.05, **p < 0.01, ***p < 0.001, ****p < 0.0001) (Fig. 10b). However, 5xFAD mice spent significantly less time interacting with the mouse compared to the WT group (p < 0.05) (Fig. 10b). Following the introduction of a novel mouse in the test for social memory, WT, 5xFAD/MMP9KO and MMP9KO mice all spent significantly more time with the novel mouse compared to the familiar mouse. Conversely, the 5xFAD mice did not show a significant preference for either the novel or the familiar mouse (Fig. 10c, d). Overall, 5xFAD mice showed a lack of social interaction and increased deficits in social memory as demonstrated by a greater disinterest in exploring the novel mouse compared to the empty cage, and the novel mouse compared to the familiar mouse, respectively. Notably, this was not observed in the 5xFAD/MMP9KO mice, which exhibited behavior akin to the WT and MMP9KO mice (Fig. 10c, d). Further analysis of this data revealed that these observed differences were driven by the male mice in each group (Fig. 11). When analyzing the males alone, the previously observed differences were more pronounced and additional differences were identified. For example, when examining the proximal zone near the cages containing the mice, male 5xFAD/MMP9KO mice spent significantly more time with the novel mouse compared to the familiar mouse (*p < 0.05) (Fig. 11d).

#### No deficits in spatial memory were identified in 5xFAD or 5xFAD/MMP9KO mice

**Fig. 12:**
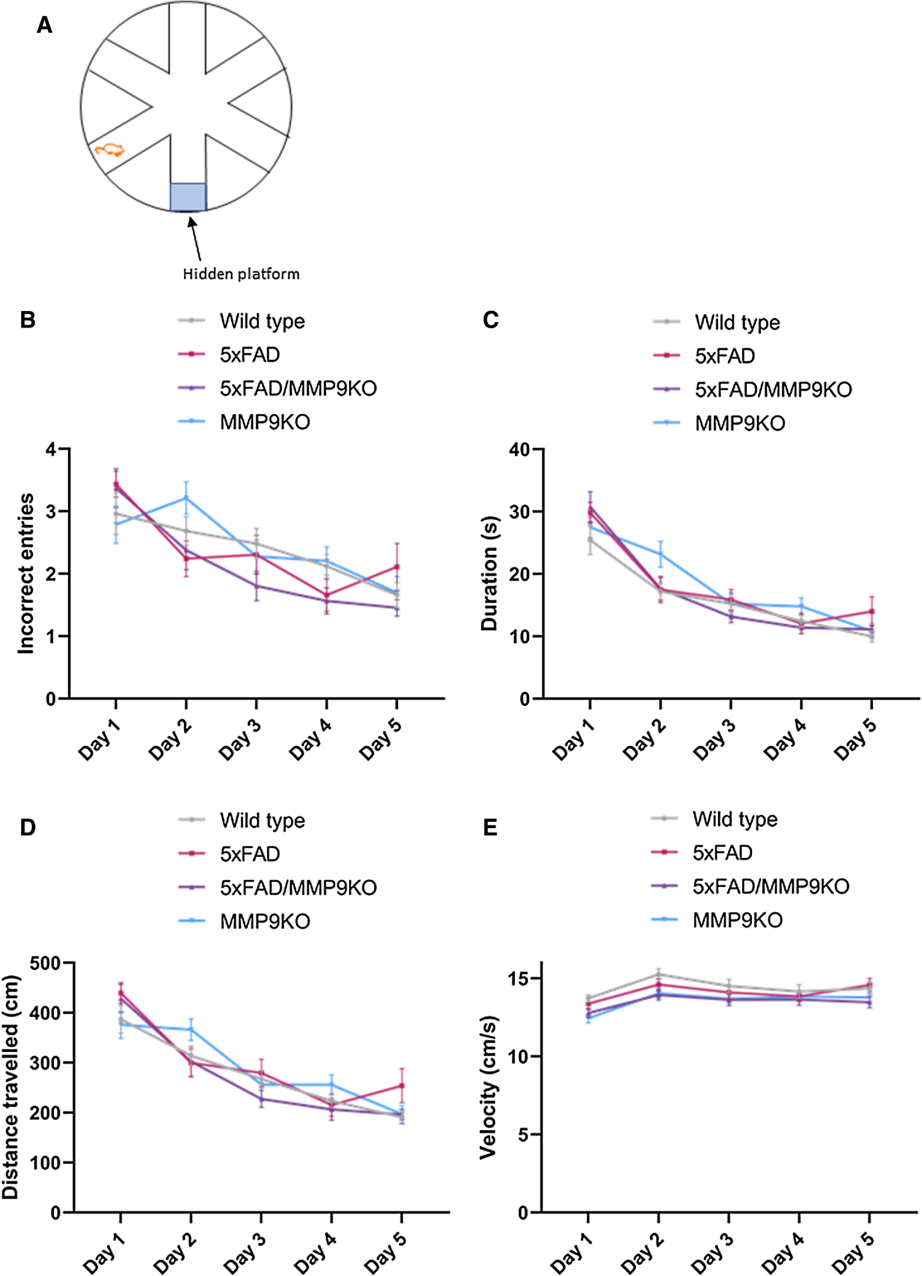
Testing spatial memory using the RAWM. Wild type, 5xFAD, 5xFAD/MMP9KO and MMP9KO mice were tested for their ability to find the hidden platform in the RAWM. **A** A schematic of the maze layout is displayed. Mice were tested in nine trials per day for 5 days. **B** The number of incorrect entries made and **C** the time taken to find the maze were recorded and analyzed. **D** The total distance travelled per trial and **E** the average velocity while swimming were also evaluated. Animals were approximately 6 months of age and values represent mean ± SEM (N = 12, 6 males, 6 females for each group). No statistical significance was identified in any of the parameters by ANOVA. *RAWM* Radial arm water maze, *WT* Wild-type, *MMP* Matrix metallopeptidase, *FAD* Familial Alzheimer’s disease, *MMP9KO* MMP9 knockout, *SEM* Standard error of the mean, *ANOVA* Analysis of variance

Following evaluation in the three-chamber test, The RAWM was used to investigate working and reference memory (Fig. 12a). Analysis of the WT, 5xFAD, 5xFAD/MMP9KO or MMP9KO mice in the RAWM revealed no differences in the number of incorrect entries made for any genotype (Fig. 12b). All genotypes made gradually fewer mistakes as the trials progressed and the time taken to reach the platform also decreased by day 5 of testing, and no differences between genotypes were identified (Fig. 12c). The number of incorrect re-entries (multiple entries into the same arm per trial) was also assessed, but after day 1 this type of error was no longer committed. When examining the distance travelled and the average velocity, no differences were detected between any of the groups (Fig. 12d, e). Additionally, there were no gender differences observed in the RAWM for any of the genotypes.

#### Amyloid levels were similar across genotypes

**Fig. 13:**
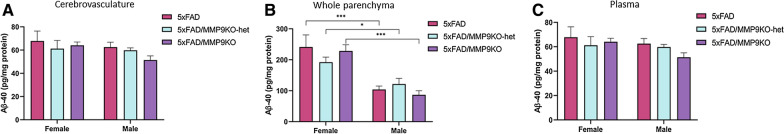
Analysis of Aβ-40 levels in the cerebrovasculature, plasma, and whole parenchyma brain fractions. Aβ-40 levels were examined in the **A** cerebrovasculature, **B** whole parenchyma, and **C** plasma of 5xFAD, 5xFAD/MMP9KO and 5xFAD/MMP9KO-het mice. Males and females were analyzed separately due to large differences in amyloid levels. Animals were approximately 6 months of age and values represent mean ± SEM (N = 12, 6 males, 6 females for each group). Statistical significance was determined by ANOVA followed by the BKY procedure. *p < 0.05, **p < 0.01, ***p < 0.001. *Aβ* Beta-amyloid, *FAD* Familial Alzheimer’s disease, *MMP* Matrix metallopeptidase, *MMP9KO* MMP9 knockout, *MMP9KO-het* MMP9KO heterozygous, *SEM* Standard error of the mean, *ANOVA* Analysis of variance, *BKY* Benjamini, Krieger, and Yekutieli

**Fig. 14:**
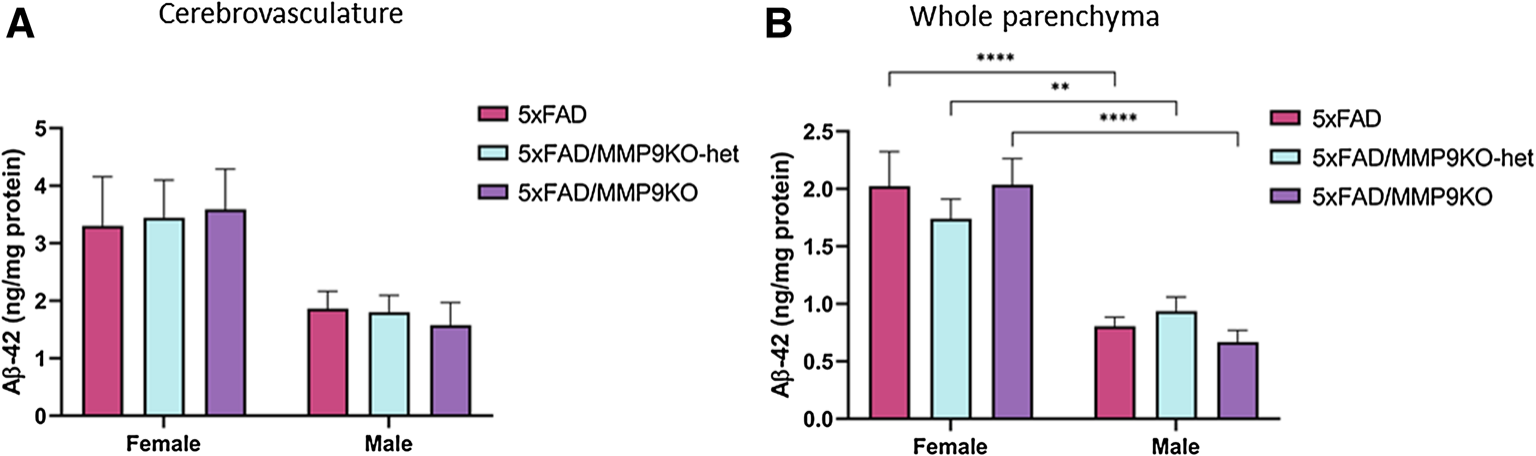
Analysis of Aβ-42 levels in the cerebrovasculature and whole parenchyma brain fractions. Aβ-42 levels were examined in the **A** cerebrovasculature, and **B** whole parenchyma of 5xFAD, 5xFAD/MMP9KO and 5xFAD/MMP9KO-het mice. Males and females were analyzed separately due to large differences in amyloid levels. Animals were approximately 6 months of age and values represent mean ± SEM (N = 12, 6 males, 6 females for each group). Statistical significance was determined by ANOVA followed by the BKY procedure. **p < 0.01, ****p < 0.0001. *Aβ* Beta-amyloid, *FAD* Familial Alzheimer’s disease, *MMP* Matrix metallopeptidase, *MMP9KO* MMP9 knockout, *MMP9KO-het* MMP9KO heterozygous, *SEM* Standard error of the mean, *ANOVA* Analysis of variance, *BKY* Benjamini, Krieger, and Yekutieli

Aβ-40 and Aβ-42 levels were evaluated in the cerebrovasculature and the brain parenchyma fractions of 5xFAD, 5xFAD/MMP9KO and 5xFAD/MMP9KO-het mice (Fig. 13, 14). WT and MMP9KO mice were excluded from analysis due to negligible Aβ levels. In addition, Aβ-40 levels were analyzed in the plasma of each mouse (Fig. 13). While no differences in Aβ-40 and Aβ-42 levels were observed in any brain fraction when comparing each genotype, amyloid levels were higher in female mice compared to male mice (*p < 0.05, **p < 0.01, ***p < 0.001) (Fig. 13, 14).

#### LDLR and LRP1 levels

**Fig. 15:**
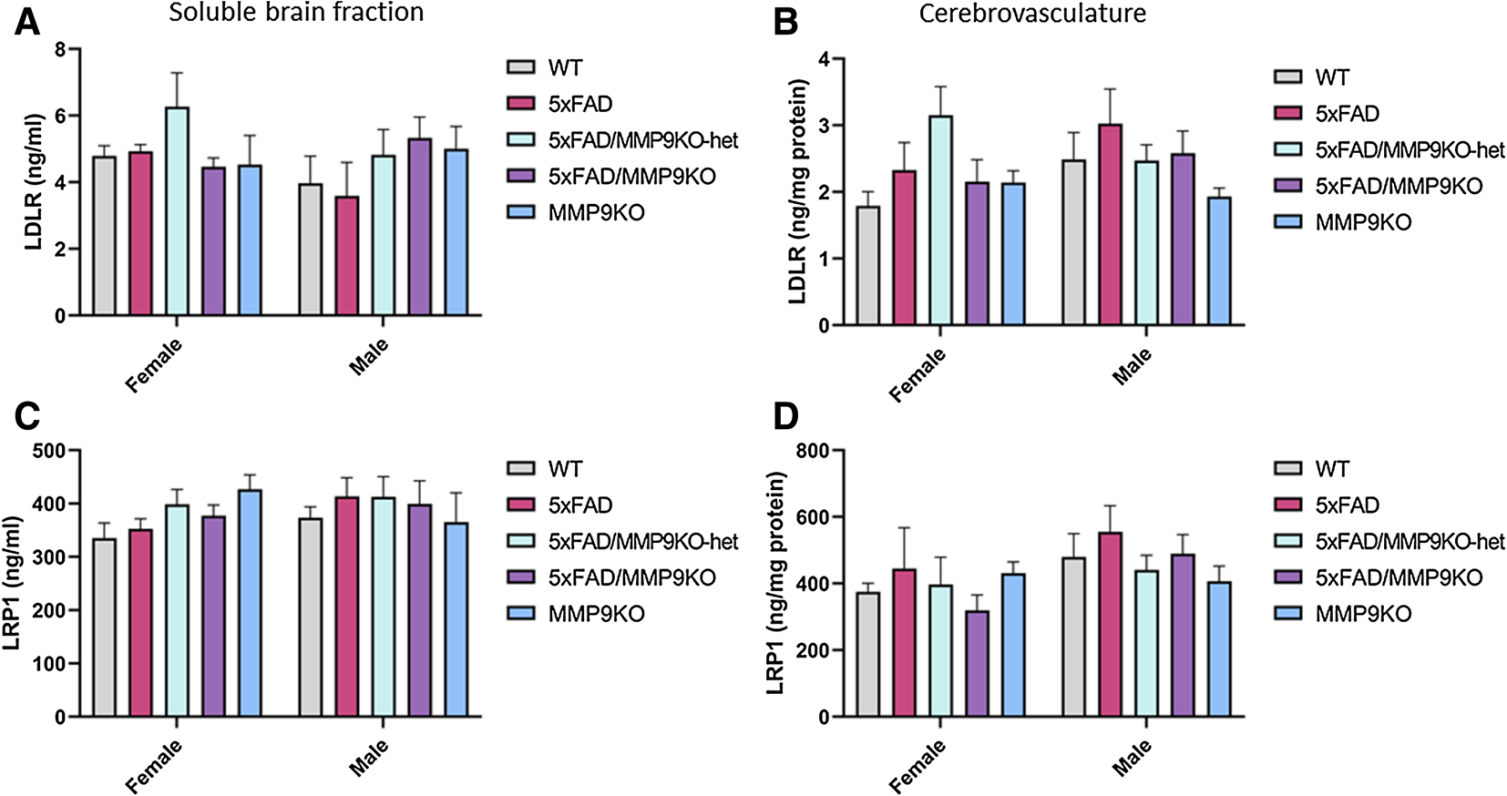
Analysis of LDLR and LRP1 levels in the cerebrovasculature and the soluble brain fraction. Levels of the **A**, **B** LDLR receptor and **C**, **D** LRP1 receptor were analyzed in the **A**, **C** soluble brain fraction and **B**, **D** the cerebrovasculature of wild type, 5xFAD, 5xFAD/MMP9KO, 5xFAD/MMP9KO-het and MMP9KO mice. Animals were approximately 6 months of age and values represent mean ± SEM (N = 12, 6 males, 6 females for each group). No statistically significant differences in LDLR or LRP1 levels were identified between any genotype in either brain fraction by ANOVA. *LDLR* Low-density lipoprotein receptor, *LRP1* Low density lipoprotein receptor-related protein 1, *FAD* Familial Alzheimer’s disease, *MMP* Matrix metallopeptidase, *MMP9KO* MMP9 knockout, *MMP9KO-het* MMP9KO heterozygous, *SEM* Standard error of the mean, *ANOVA* Analysis of variance

LDLR and LRP1 levels were examined in WT, 5xFAD, 5xFAD/MMP9KO, 5xFAD/MMP9KO-het and MMP9KO mice to determine the effect of MMP9 gene deletion on lipoprotein receptor shedding (Fig. 15). No differences in these receptors were detected between any genotype in the soluble brain fraction (Fig. 15a, c) or isolated cerebrovasculature (Fig. 15b, d). There were no gender differences observed in the analysis of LDLR or LRP1 for any of the genotypes.

## Discussion

Elevated MMP9 levels are apparent in a variety of neurological disorders where they have been shown to perpetuate disease progression [4–7, 50–54]. As our prior studies showed MMP9 levels and activity are elevated in the presence of the apoE4 isoform in AD [18], and MMP9 inhibition can facilitate Aβ elimination across the BBB, the present studies evaluated the effect of MMP9 inhibition in E4FAD mice. Moreover, to further validate the role of MMP9 in AD pathogenesis and neurobehavioral dysfunction, we examined influence of MMP9 in AD by crossing 5xFAD mice with MMP9KO mice. Behavioral and psychological symptoms are common in AD and can significantly impact social interactions, leading to social withdrawal [60–64]. While mice are generally social animals [65], in the current studies the E4FAD mice did not display a preference for a mouse compared to the empty cage, indicative of reduced sociability, as measured using the three-chamber test. Similarly, though the 5xFAD mice did spend significantly more time with the mouse than the empty cage, it did so to a lesser degree than the WT mice, consistent with previous studies demonstrating reduced sociability in this mouse AD model, which progresses from 3 months of age onward [66]. The reduced social interaction by the 5xFAD mice was not apparent in the 5xFAD/MMP9KO mice, suggesting that removal of the MMP9 gene in this mouse AD model beneficially impacted sociability. Furthermore, when a novel mouse was introduced, the WT, MMP9KO, and 5xFAD/MMP9KO mice all spent significantly more time with the novel mouse versus the familiar mouse, however the 5xFAD mice did not, indicating an impairment in social recognition memory, in line with previous reporting for this mouse AD model [67]. Notably, this impairment was not observed when the MMP9 gene was absent. The CA2 region of the hippocampus has been shown to be crucial for sociocognitive memory processing [68] and the hippocampus shows a considerable elevation in Aβ levels in 6 month-old 5xFAD mice [69].

As mentioned above, our previous research demonstrated that inhibition of MMP9 mitigated Aβ-induced lipoprotein receptor shedding in the brains of apoE4 mice in addition to increasing the transit of intracranially injected Aβ from the brain to the periphery [17]. However, this does not appear to be the primary mechanism driving the improved social memory in the 5xFAD/MMP9KO mice since Aβ-40 and Aβ-42 levels in the parenchyma, cerebrovasculature, and Aβ-40 levels in the plasma remained constant across genotypes. Moreover, the levels of the lipoprotein receptors were unchanged between the different genotypes, suggesting there was no discernible impact on LRP1 and LDLR shedding when MMP9 was genetically absent. While removing the MMP9 gene in the 5xFAD mice did not result in obvious changes in Aβ disposition, it may have prevented the contribution of MMP9 to Aβ-induced cognitive deficits [10]. Mizoguchi et al. demonstrated that the injection of Aβ increases MMP9 expression and that this increase is associated with the development of cognitive impairment and neurotoxicity [10]. Recognition memory, as measured by the novel object recognition test, was impaired in Aβ-injected WT mice, but not MMP9KO mice or WT mice treated with an MMP inhibitor [10], coinciding with the current studies using the three-chamber test. Like other mouse models of AD, 5xFAD mice display increased levels of MMP9 compared to WT mice [70, 71], which could be contributing to the observed deficits in social recognition memory in these mice. Thus MMP9 could be a target to improve social memory in AD or potentially other disease conditions.

While it was anticipated that MMP9 modulation would alter brain Aβ levels by facilitating lipoprotein receptor transit across the BBB, as demonstrated in our prior work [17], removing MMP9 may prevent other harmful actions of this enzyme and could explain the differences observed between the 5xFAD and the 5xFAD/MMP9KO mice. For instance, the degradation of matrix proteins in the vascular basal lamina by MMP9, leading to BBB disruption, has been suggested to contribute to the brain damage that occurs following cerebral ischemia [72–74], effects that can be attenuated through MMP9 inhibition [25, 26]. Alternatively, the degradation of laminin or other matrix proteins by MMP9 throughout the brain may disrupt cell–matrix interactions and play a role in neuronal cell death [24], which could be attenuated through MMP9 modulation. In fact, inhibition of MMP9 has been shown to reduce tissue damage, neutrophil infiltration, oxidative stress and degenerating neurons [7, 25, 26], all factors that could contribute to the impaired social memory in the 5xFAD mice [36, 75–79].

MMPs, in particular MMP9, have been suggested to have a generalized role in the maintenance of long-term potentiation (LTP) and nonpathological synaptic plasticity in adult brains [80–83]. In previous studies, MMP9 inhibition led to the disruption of late-phase LTP [80–83], however, excessive MMP9 activity is deleterious to cells and MMP9 inhibition can enhance LTP when MMP9 activity is excessive and prolonged [84]. Under normal conditions, MMP9 activity is strictly regulated and the transient proteolytic activity of the enzyme required for structural remodeling is focal and quickly terminated following its completion. Conversely, under pathological conditions, MMP9 activity is widespread and sustained, leading to abnormal synaptic plasticity and impairments in cognitive function [84, 85], which may describe the deficits in social recognition memory in the 5xFAD animals that were reversed in the 5xFAD/MMP9KO mice.

In terms of sex, the effects of MMP9 modulation on social interaction and social memory in the 5xFAD group were largely driven by the male mice, which tend to show inherently less pathology compared to female mice in this AD model [36, 86]. This type of trend has been observed in clinical studies, where therapies appear to be more effective when administered early in the disease process and/or the pathology is less advanced [87, 88]. Akin to our studies, prior in vivo work observed that male AD mice showed a markedly reduced social interaction compared to female AD mice at 12 months of age, however these effects were reversed at 18 months of age as the female AD mice demonstrated less social interaction than male AD mice [124]. In contrast to social recognition memory, spatial recognition memory was not impacted by MMP9 deletion or inhibition, however, male E4FAD mice treated with SB-3CT located the hidden platform slightly faster than the vehicle-treated mice over the course of the 5 days. This finding was not statistically significant, possibly owing to the reduced number of mice in this subgroup analysis (N = 5) [89, 90]. Spatial disorientation is frequently observed in AD with patients displaying impaired visuospatial memory [91–93]. Spatial recognition memory has been previously shown to be impaired in 4-month-old E4FAD mice using the two-trial Y-maze and the Morris Water Maze [94], however, in the RAWM used in our studies, the control E4FAD mice continued to learn each day, making few mistakes by day 5 and quickly finding the platform. Likewise, the performance of the 5xFAD mice at 6 months of age was similar to the WT mice as they did not exhibit deficits in spatial memory. Deficits in spatial memory and recognition memory are both initial symptoms of AD [95], though spatial memory typically presents earlier in the spectrum of cognitive impairment than recognition memory [67, 96–99]. In contrast, the 6-month-old 5xFAD mice in the current studies displayed impairment of social recognition memory but not spatial memory. While memory impairment in this 5xFAD mouse strain has been detected as young as 1 month of age through the Morris water maze [100, 101], others have found that 5xFAD mice display normal spatial memory function in this test until 7 months of age [102] or even up to 12 months of age [103]. Likewise, recognition memory has been shown to emerge at 4 months of age [103] in one study and at 9 months of age in another [67]. These findings indicate behavioral observations can vary considerably from one experiment to another and memory impairment can be specific to context, modality, and/or environment [66]. Because the E4FAD and the 5xFAD made few errors by day 5, it is difficult to discern whether the SB-3CT treatment or the MMP9 gene knockout had any impact on spatial memory in these studies. Future studies may need to assess the effect of both approaches in older mice where the deficits in spatial memory are more apparent, while accounting for potential differences in gender.

It has been demonstrated that 5xFAD mice display reduced anxiety in the EPM compared to WT mice, as measured by increased time spent in the open arms compared to the closed arms [75, 104, 105]. In our studies, pharmacological and genetic modulation of MMP9 in the AD models increased the time spent in the closed arms of the EPM, though only the pharmacological studies reached statistical significance. In the OFT, the reduced time spent in the outer areas of the arena and increased time in the middle of the arena by the 5xFAD mice compared to all other genotypes indicates reduced anxiety or tendency toward disinhibition. Disinhibition is one of the neuropsychiatric symptoms seen in AD patients, manifesting as impulsive behavior and the disregard for danger [106–108]. The removal of MMP9 appeared to mitigate the anxiolytic behavior in the 5xFAD mice as the behavioral pattern of the 5xFAD/MMP9KO mice in the OFT was similar to the WT mice. It is also necessary to analyze locomotor behavior in these tests since anxiety measures can be confounded by levels of activity [109]. The increased locomotor activity in the 5xFAD mice has been previously documented and attributed to alterations in neurotransmitter levels [67, 110] in addition to decreased anxiety [111]. In the present studies, 5xFAD/MMP9KO mice had significantly lower locomotor activity compared to the 5xFAD mice as measured by distance travelled and average velocity, again suggesting MMP9 may be influencing anxiety-related behavior. In fact, MMP9 has been associated with an increased susceptibility to anxiety disorders [112] as well as showing elevated levels in other psychiatric illnesses [113–115]. Human genomic and proteomic studies have revealed that aberrant inflammatory responses, gene regulation, and synaptic plasticity are major players underlying neuropsychiatric disorders. As MMP9 has been implicated in both the inflammatory response and synaptic plasticity, its contribution to the development of psychiatric symptoms may be mediated through these processes [115]. However, further research needs to be conducted to understand the precise mechanisms that are involved.

## Limitations

Overall, while we did observe significant differences in social memory between the WT, 5XFAD, and 5XFAD/MMP9KO mice, namely the 5xFAD mice exhibited social memory deficits compared to both the WT and the 5XFAD/MMP9KO mice, these effects were relatively modest. It is anticipated, these differences would be more apparent with increased age and/or altered brain pathology in the 5xFAD mice. The studies conducted by Mizoguchi et al., point to the direct involvement of MMP9 in Aβ-induced cognitive deficits as MMP9 inhibition and knockout prevented the impairment of social recognition memory [116]. Therefore, a more detailed analysis of specific changes in the Aβ population following MMP9 removal in our studies, for instance shifts in the brain location, forms, or toxic species of Aβ, may be necessary to understand the influence of MMP9 on behavioral performance. Lastly, in the current studies all animals underwent the same series of behavioral tests, and thus can be reasonably compared across groups. However, it is worth noting that animals exposed to multiple behavioral paradigms in a relatively short period of time can develop a training effect, in which participation in one task could influence the response in a subsequent task.

Removing the MMP9 gene in the 5xFAD mice did result in more pronounced differences in neurobehavior compared to pharmacological inhibition of MMP9 in the E4FAD animals, which may be due to several factors. Most notable, in the 5XFAD/MMP9KO animals, MMP9 is mitigated earlier (from birth), more extensively (complete removal), and for a longer period of time (lifespan) than the pharmacological studies. That having been said, the MMP9KO studies occurred in the presence of murine apoE, as opposed to the human apoE isoforms. ApoE-driven AD pathologies differ from pure amyloid pathologies [117, 118] and our prior work indicates apoE can regulate MMP9 disposition in the brain, and the apoE4 isoform in particular is associated with elevated MMP9 levels and activity [18]. Therefore, a limitation of the genetic knockout studies may be the absence of the apoE4 isoform which, as mentioned, exacerbates MMP9 activity and is closely associated with AD pathogenesis. It is also worth noting that the removal of a gene at birth may not entirely reflect its role in a disease process, particularly for age-related disorders, as adaptation or compensation by other genes may occur prior to disease onset. As such, the use of a conditional knockout model or adeno-associated viruses (AAV) may provide a more accurate representation of the impact of MMP9 in AD and the potential for therapeutic intervention.

The altered anxiety in the E4FAD mice indicates that treatment with SB-3CT may be efficacious, however, due to limitations in detection, the proteolytic activity of MMP9 in the brain could not be measured and we could not confirm target engagement. This finding is consistent with other reports owing to the rapid degradation of the activated MMP9 enzyme in vivo [24]. The mechanism of action of SB-3CT is to modulate MMP9 activity, not MMP9 expression (119). As expected, total MMP9 levels in the E4FAD brains remained unaltered, emphasizing that MMP9 expression is not a good indicator of target engagement when assessing SB-3CT. Owing to this limitation, it is not certain whether the observed effect on anxiety in the current studies was caused by the inhibition of MMP9 activity or another effect of the drug. In prior reporting, the same dose of SB-3CT used in our studies showed significant reductions in MMP9 activity in the brain using a treatment paradigm more acute than that used in the present studies [6, 31]. Thus, it seems likely MMP9 activity was inhibited in the current studies, but a more chronic treatment paradigm may be necessary to overcome the AD phenotype. Moreover, intervention at an earlier stage may be required since the SB-3CT treatment in the current studies began at 4-months of age in the E4FAD mice, a time at which brain pathology is already apparent [102, 120]. Additionally, both MMP9 and apoE4 have been implicated in early AD pathology before the onset of cognitive impairment [55–59], so therapeutic approaches targeting MMP9 may require early administration to see the greatest benefits.

The inherent limitations of transgenic AD mouse models when investigating the underlying mechanisms and potential treatments for sporadic AD must also be noted. 5xFAD mice are based on early-onset AD and co-express five FAD mutations, resulting in excessively high levels of Aβ-42 at a relatively early age, and therefore do not convincingly represent the chronic age-related progression of sporadic AD pathology, which represents > 97% of the AD population [121, 122]. That said, while AD pathology is well established at 6 months of age in the AD models used in the current studies, and age is a prominent factor in AD pathogenesis, the pathological or behavioral deficits may have been more apparent (and perhaps the impact of MMP9 modulation) if these animals were interrogated at a more advanced age (> 12 months). The inability to recapitulate sporadic AD in animal models due to the complex and heterogenous nature of the disease has hindered the development of effective treatments (Additional file 1: Figure S1). The use of more accurate animal models of late-onset AD would facilitate the drug development process in AD and identify more appropriate drug candidates for clinical testing. With respect to the current studies, using more representative models of sporadic AD would likely improve investigation into the role of MMP9 in AD pathogenesis and cognitive dysfunction, and its potential as a therapeutic target for this disorder.

## Conclusions

MMP9 levels in the brain are elevated in a variety of neurological disorders, including AD, and contribute to disease formation and progression. In the current studies, MMP9 modulation in AD animals improved sociability and social recognition memory, particularly in male mice, in addition to reducing anxiety, while spatial learning and memory was unaffected. In our prior work MMP9 inhibition increased Aβ elimination across the BBB, however, modulating MMP9 in the present studies did not alter Aβ tissue levels in AD animals. As such, MMP9 appears to mediate certain neurobehavioral deficits in AD, such as anxiety and social recognition memory, but these effects are independent of Aβ levels in the brain. While therapeutic strategies targeting MMP9 could improve some aspects of neurobehavioral dysfunction in AD, additional studies are needed to better understand the role of MMP9 in neurological disease.

## Supplementary Information

**Additional file 1**: **Figure S1.** MMP9 levels in spleen samples from SB-3CT-and placebo-treated E4FAD mice. Image depicts the full gel zymogram showing bands for proMMP9 in SB-3CT-treated and control (placebo-treated) animals. Lane 1: Naïve E4FAD mouse from another study; Lanes 2-6: SB-3CT-treated E4FAD mice; Lanes 7-10: Placebo-treated E4FAD mice; Lanes 11-14: Naïve E3FAD mice from another study.

## Data Availability

The datasets used and/or analyzed during the current study are available from the corresponding author on reasonable request.

## Abbreviations

AD: Alzheimer’s disease
ANOVA: Analysis of variance
APOE: Apolipoprotein E
ApoE-TR: ApoE targeted replacement
APP: Amyloid precursor protein
Aβ: Amyloid beta
BBB: Blood brain barrier
BCA: Bicinchoninic acid
BKY: Two-stage step-up method of Benjamini, Krieger and Yekutieli
CSF: Cerebrospinal fluid
DMSO: Dimethyl sulfoxide
EDTA: Ethylenediaminetetraacetic acid
ELISA: Enzyme linked immunosorbent assay
EPM: Elevated plus maze
FAD: Familial Alzheimer’s disease
HBSS: Hanks Balanced Salt Solution
LDLR: Low-density lipoprotein receptor
LOAD: Late-onset Alzheimer’s disease
LRP1: Low density lipoprotein receptor-related protein 1
LTP: Long-term potentiation
MMP: Matrix metallopeptidase
MMP9KO: MMP9 knockout
M-PER: Mammalian Protein Extraction Reagent
OFT: Open field test
PEG: Polyethylene glycol
PMSF: Phenylmethylsulphonyl fluoride
PS1: Presenilin 1
RAWM: Radial arm water maze
SB-3CT: 2-[[(4-Phenoxyphenyl)sulfonyl]methyl]thiirane
SEM: Standard error of the mean
WT: Wild type
