# Supplementary material for: MMP9 modulation improves specific neurobehavioral deficits in a mouse model of Alzheimer’s disease
Source: BMC Neurosci. 2021 May 25;22:39. doi: 10.1186/s12868-021-00643-2 (PMC8152085; doi:10.1186/s12868-021-00643-2)
Supplement: Supplementary file 1 — Additional file 1: Figure S1. MMP9 levels in spleen samples from SB-3CT-and placebo-treated E4FAD mice. Image depicts the full gel zymogram showing bands for proMMP9 in SB-3CT-treated and control (placebo-treated) animals. Lane 1: Naïve E4FAD mouse from another study; Lanes 2-6: SB-3CT-treated E4FAD mice; Lanes 7-10: Placebo-treated E4FAD mice; Lanes 11-14: Naïve E3FAD mice from another study. [file 12868_2021_643_MOESM1_ESM.pdf]

MMP9 modulation improves specific neurobehavioral deficits in a mouse model of Alzheimer's disease

Charis Ringland[a,b], Jonas Elias Schweig[a], Maxwell Eisenbaum[a,b], Daniel Paris[a], Ghania Ait-Ghezala[a,b], Michael Mullan[a,b], Fiona Crawford[a,b,c], Laila Abdullah[a,b,c], Corbin Bachmeier[a,b,d] *

[a]The Roskamp Institute, 2040 Whitfield Avenue, Sarasota, FL, 34243, USA.

[b]The Open University, Milton Keynes, UK.

[c]James A. Haley Veterans' Hospital, Tampa, FL, USA.

[d]Bay Pines VA Healthcare System, Bay Pines, FL, USA.

*Corresponding author:

Corbin Bachmeier

2040 Whitfield Avenue

Sarasota, Florida 34243, USA

Phone : 941.752.2949

Fax : 941.752.2948

cbachmeier@roskampinstitute.org

Supplementary Information

Supplementary Figure S1

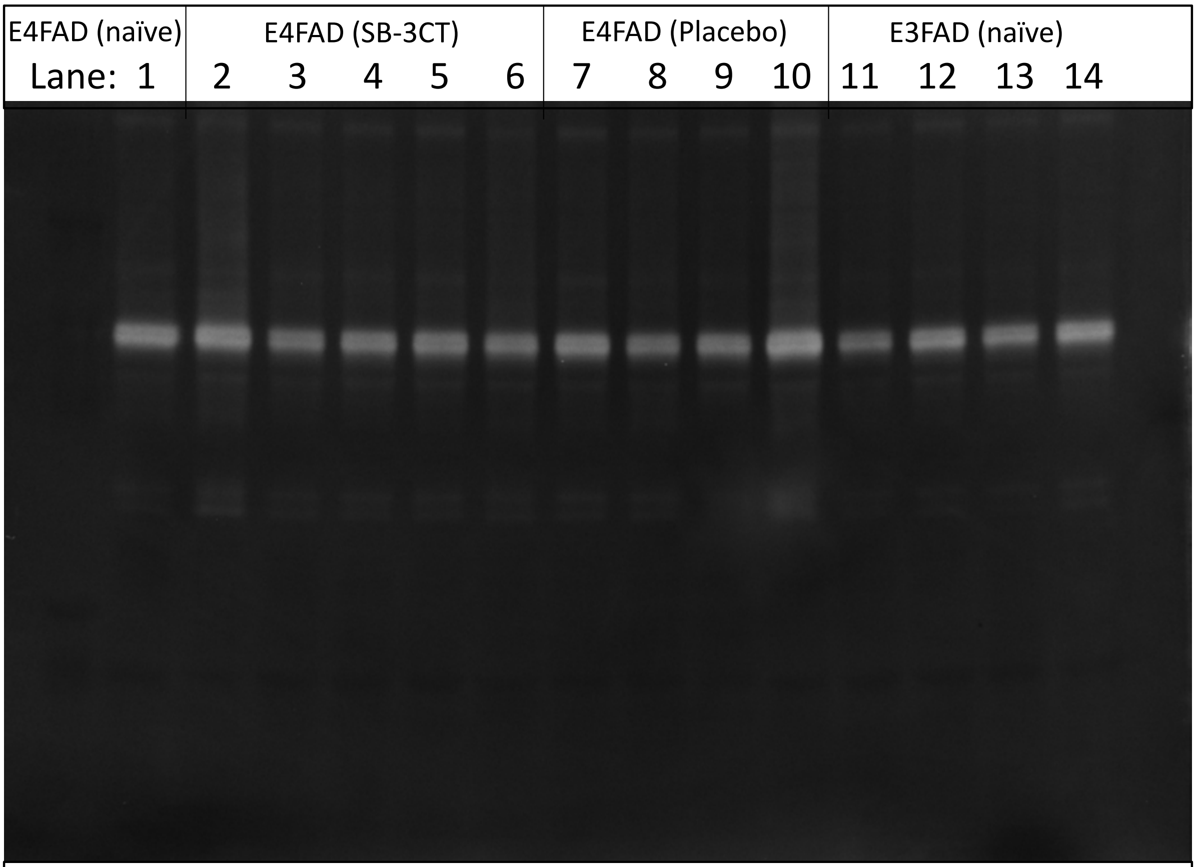

Supplemental Figure S1: MMP9 levels in spleen samples from SB-3CT- and placebo-treated E4FAD mice. Image depicts the full gel zymogram showing bands for proMMP9 in SB-3CT-treated and control (placebo-treated) animals. Lane 1: Naïve E4FAD mouse from another study; Lanes 2-6: SB-3CT-treated E4FAD mice; Lanes 7-10: Placebo-treated E4FAD mice; Lanes 11-14: Naïve E3FAD mice from another study.
